# Vibrational Energy Transfer in CO+N_2_ Collisions: A Database for V–V and V–T/R Quantum-Classical Rate Coefficients

**DOI:** 10.3390/molecules26237152

**Published:** 2021-11-25

**Authors:** Qizhen Hong, Massimiliano Bartolomei, Cecilia Coletti, Andrea Lombardi, Quanhua Sun, Fernando Pirani

**Affiliations:** 1 State Key Laboratory of High Temperature Gas Dynamics, Institute of Mechanics, Chinese Academy of Sciences, Beijing 100190, China; hongqizhen@imech.ac.cn (Q.H.); qsun@imech.ac.cn (Q.S.); 2 School of Engineering Science, University of Chinese Academy of Sciences, Beijing 100049, China; 3 Instituto de Física Fundamental—CSIC, C/Serrano 123, 28006 Madrid, Spain; maxbart@iff.csic.es; 4 Dipartimento di Farmacia, Università G. d’Annunzio Chieti-Pescara, Via dei Vestini, I-66100 Chieti, Italy; 5 Dipartimento di Chimica, Biologia e Biotecnologie, Università di Perugia, Via Elce di Sotto, I-06183 Perugia, Italy; andrea.lombardi@unipg.it (A.L.); fernando.pirani@unipg.it (F.P.)

**Keywords:** vibrational energy transfer, inelastic scattering, potential energy surface, rate coefficients database, quantum-classical nuclear dynamics, quasi-classical trajectories

## Abstract

Knowledge of energy exchange rate constants in inelastic collisions is critically required for accurate characterization and simulation of several processes in gaseous environments, including planetary atmospheres, plasma, combustion, etc. Determination of these rate constants requires accurate potential energy surfaces (PESs) that describe in detail the full interaction region space and the use of collision dynamics methods capable of including the most relevant quantum effects. In this work, we produce an extensive collection of vibration-to-vibration (V–V) and vibration-to-translation/rotation (V–T/R) energy transfer rate coefficients for collisions between CO and N${}_{2}$ molecules using a mixed quantum-classical method and a recently introduced (A. Lombardi, F. Pirani, M. Bartolomei, C. Coletti, and A. Laganà, Frontiers in chemistry, 7, 309 (2019)) analytical PES, critically revised to improve its performance against ab initio and experimental data of different sources. The present database gives a good agreement with available experimental values of V–V rate coefficients and covers an unprecedented number of transitions and a wide range of temperatures. Furthermore, this is the first database of V–T/R rate coefficients for the title collisions. These processes are shown to often be the most probable ones at high temperatures and/or for highly excited molecules, such conditions being relevant in the modeling of hypersonic flows, plasma, and aerospace applications.

## 1. Introduction

The kinetic modeling of gases is a relevant issue for the understanding and for the simulation of complex gaseous environments covering Earth and planetary atmospheres [1,2], combustion processes, plasma chemistry [3], and hypersonic aerodynamics [4,5], just to mention a few. In many such environments the population of the molecular vibrational states often strongly deviates from the Boltzmann distribution and the detailed description of the gas behavior relies on the knowledge of non-equilibrium energy transfer processes occurring upon collisions. More specifically, the modeling of bulk processes implies the solution of a master equation where the loss/gain of vibrational quanta of energy for molecular species in any initial vibrational quantum state should be included. This means that a very large number of rate coefficients for vibration-to-translation/rotation (V–T/R) and vibration-to-vibration (V–V) energy exchange processes, the most effective for determining the evolution of the vibrational distribution, needs to be known with high accuracy in a large range of temperature.

Unfortunately, experimental measures of such quantities are restricted to a very small number of V–V or V–T/R processes, often involving only low-lying vibrational quantum states. As a consequence, most rates are derived from theoretical calculations, which should be not only reliable but also computationally fast, because of the wealth of processes to be considered. Quasi-classical trajectories (QCT) dynamical treatments are often used, where classical Hamilton equations of motion are propagated in time. In this case, due to the intrinsic quantum nature of the vibrational energy exchange process, a quantum treatment would be highly desirable. However, full quantum mechanical calculations often remain prohibitive for the description of inelastic scattering in four-body systems, because of their computational burden. Mixed quantum-classical (QC) methods, on the other hand, have the advantage of maintaining the computational (and conceptual) simplicity of QCT calculations, while introducing a quantum description for those degrees of freedom which are expected to show a more pronounced quantum behavior in the investigated conditions [6]. Specifically, for processes involving the transfer of vibrational energy quanta, the diatom vibrations in the quantum-classical method are described by solving the corresponding time-dependent Schrödinger equation, whereas for the remaining degrees of freedom the classical equations of motion are propagated under the influence of an effective potential, obtained as the quantum expectation value [7,8,9]. Such an approach has proved to provide accurate results in a wide temperature range with approximately the same numerical effort of QCT methods.

For this reason, the quantum-classical (QC) approach, sometimes also referred to as semiclassical, has been used over the years for the description of inelastic scattering in a variety of diatom–diatom systems: N${}_{2}$+N${}_{2}$ [10,11,12,13], CO+CO [14,15], N${}_{2}$+CO [11,16,17,18], O${}_{2}$+O${}_{2}$ [19,20,21], etc. leading, in some cases, to the creation of large databases for V–V rate coefficients. Databases for V–T/R rate coefficients are instead much more difficult to find, and in fact, the numerical determination of such quantities is often limited to processes involving the loss of one quantum of energy of the first vibrationally excited states. The accurate computation of V–T/R coefficients is computationally more demanding than that of V–V rates: V–T/R rate coefficients are more sensitive to long-range interactions and require larger initial separation distances of the diatoms, up to 70–80 Å, increasing the necessary computation time. Therefore, V–T/R rate coefficients are still often inferred through first-order approaches, like the Schwartz–Slawsky–Herzfeld (SSH) theory [22], which neglect multiquantum processes and are based on the short-range potential only, leading to approximate values, particularly in the very high or very low-temperature regimes, although the use of scaling procedures can improve their performance. V–T/R rates are believed to play a less important role than V–V ones, particularly when the latter involve nearly resonant energy exchange, but this is usually true in the low-temperature regime only. High-temperature conditions and high vibrational quantum numbers greatly favor V–T/R processes and, as we highlighted in recent investigations on N${}_{2}$-N${}_{2}$[13] and O${}_{2}$+O${}_{2}$ [21], they can even become the most favorable events, heavily contributing to the overall molecular vibrational distribution. Such temperature regimes are those characterizing, for instance, hypersonic flows around re-entry vehicles. The temperature behind the shock wave is high enough for stimulating inelastic internal energy exchanges (V–T/R, V–V, V–E, and so on) and chemical reactions, and accurate modeling of those physical phenomena is necessary to predict the surface heat flux of the vehicle.

The present study aims to provide a large database of V–V and V–T/R rate coefficients for collisions between molecular nitrogen N${}_{2}$ and carbon monoxide CO, calculated through the mixed quantum-classical method [7,8,9,13,14,21]. A detailed database for V–V processes, including some multiquantum transitions, calculated through the QC method, is available up to T = 2900 K [11]. Here we extend the investigation to consider temperatures up to 7000 K and higher vibrational states. To the best of our knowledge, there are no databases for N${}_{2}$+CO V–T/R transitions, even if N${}_{2}$+CO mixtures can be found in many different environments, where high temperatures can be reached and V–T/R processes are essential for their characterization. Indeed, N${}_{2}$ and CO are both important components of many atmospheres of our solar system (e.g., Titan, Triton, Pluto, and Mars [23]) and extrasolar planetary systems. Furthermore, CO quite obviously plays an important role in combustion chemistry and in CO${}_{2}$ plasma. Because of the aforementioned difficulty in their determination, presently available repositories for combustion chemistry or astrochemical data contain structural and dynamical values which are often derived from simple extrapolations or oversimplified computations. For this reason we believe that the accurate calculation of V–V and of the unprecedented V–T/R rates for a large variety of initial molecular vibrational states and a wide temperature range, carried out in an internally consistent way on the same potential energy surface, might represent a step forward in the kinetic modeling of a wealth of gaseous environments.

One of the main ingredients to obtain reliable results in the treatment of collisional dynamics is the use of an accurate potential energy surface (PES), capable of describing, in detail, intermolecular energy at long and short range for all possible reciprocal orientations of the diatoms. Reduced dimensionality PESs based on high-level ab initio calculations (at CCSD(T) level coupled to basis sets of quadrupole-ζ quality) were recently published to describe the N${}_{2}$-CO system [24,25,26]. They have led to a very accurate determination of the roto-vibrational spectrum of the van der Waals complex, but they cannot be used to describe vibrational energy exchange processes with the same confidence, because the intramolecular distance in one or both diatoms is kept frozen. Furthermore, as shown for N${}_{2}$-N${}_{2}$ collisions [27,28], in general, ab initio based potentials might be inaccurate when very large initial separation distances of the colliding partners are required: the number of ab initio points needed even for an approximate description of all long-range configurations is still prohibitive and interpolation procedures might produce spurious effects [29]. On the other hand, analytical and/or semiempirical potentials, often simply constructed as a sum of repulsive short-range and attractive long-range components of the interaction potential, have been proposed [11,17] and, provided that all interaction regions (long-range, interaction wells, and repulsive walls) are appropriately taken into account, they are found to reproduce V–V and V–T/R rate coefficients quite effectively.

We recently introduced a full dimensional analytical surface [18] by using a well-established semiempirical approach and tested it to reproduce experimental values, like the second virial coefficients, and against accurate ab initio energies. In the same paper preliminary calculations for selected V–V processes, for which experimental rate coefficients are available, were carried out, which showed an overall good qualitative agreement in a wide temperature range, with some quantitative discrepancies in the low temperature regime.

In order to produce an accurate database, in the present study we start by improving the above PES, according to a procedure we successfully applied in the case of N${}_{2}$-N${}_{2}$ [13], O${}_{2}$-O${}_{2}$[21], and N${}_{2}$-O [30] systems. Indeed, one of the main advantages of this analytical formulation, which is far from being a simple fit to ab initio or experimental data, is the possibility of modulating its behavior by modifying the physically meaningful parameters in a limited range. This permits to keep an eye on the physics of the process and on the relevant configurations controlling the dynamics of different phenomena.

The paper is therefore organized as follows. In Section 2 a short description of the CO+N${}_{2}$ PES is given, together with its comparison against experimental and ab initio data and its subsequent improvement against experimental results. A comparison between predictions of QCT and QC methods is also analyzed in this Section. Section 3 reports a database and a critical discussion of the V–V and V–T/R rate coefficients calculated with the improved PES, aimed to identify the most relevant collisional events taking place at different temperature conditions. Concluding remarks are given in Section 4.

## 2. Potential Energy Surface

The formulation of the full PES is essentially the same one of [18], which will be briefly described in the following. The approach, which has been successfully applied to O${}_{2}$-O${}_{2}$ [21], N${}_{2}$-N${}_{2}$ [13,31], N${}_{2}$-H${}_{2}$ [32], and CO${}_{2}$-CO${}_{2}$ [33], expresses the potential function parameters in terms of the binding energy and internal molecular structure (intramolecular part) and of properties like charge distributions and polarizabilities (intermolecular part).

The overall interaction V of the diatom–diatom system is thus described as a sum of the intramolecular ($V_{intra}$) and intermolecular ($V_{inter}$) interaction components: $V_{intra}$ is formulated using a Morse potential energy function $D_{e}\left(t^{2}-2t\right)$, in which $D_{e}$ is the dissociation energy of the diatomic molecule, $t=\exp\left[-\beta \left(r-r_{e}\right)\right]$ and *r* is the internuclear diatomic distance (with $r_{e}$ being its equilibrium value). The set of Morse parameters derived from spectroscopic data [34] is shown in Table 1. The same set of parameters is used to define the Morse function in the quantum-classical calculations.

### 2.1. The Improved Lennard–Jones Formulation

**Figure 1:**
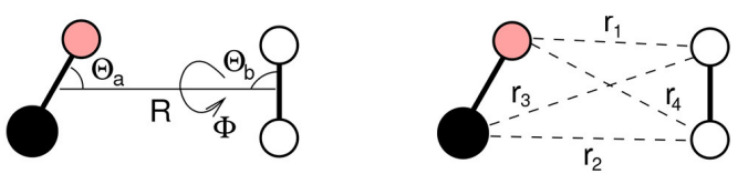
Schematic diagram of CO-N${}_{2}$ dimer with the coordinates needed to define the intermolecular interaction contributions in Equation (1).

The intermolecular ($V_{inter}$) interaction component is represented as the sum of two main contributions:

(1) $$V_{inter}=V_{vdW}+V_{elect},$$

where $V_{vdW}$ and $V_{elect}$ represent the van der Waals (size repulsion plus dispersion-attraction) and the electrostatic interaction components, respectively. Both terms depend on the four-body Jacobi coordinates: the distance *R* between the centers of mass of the interacting partners, and the angles $\Theta_{a},\Theta_{b},\Phi$, which describe the relative orientation of the two diatoms (see Figure 1).

The van der Waals term, determined by the combination of exchange-repulsion with dispersion attraction, is expressed as a sum of the non-covalent contributions:

(2) $$V_{vdW}\left(R,\Theta_{a},\Theta_{b},\Phi \right)=\sum_{i=1}^{4}V_{vdW}^{i}\left(r_{i}\right),$$

where $r_{i}$ is the distance between atoms of different interacting molecules and the summation runs over all four atom pairs of the CO-N${}_{2}$ dimer (Figure 1). The adopted formulation is capable to properly account for the variation of the interaction anisotropy with the atom–atom distance [35]. The explicit form of $V_{vdW}^{i}$ term is obtained by using an improved Lennard–Jones (ILJ) potential [36] depending on few parameters related to atomic or molecular properties of the interacting partners [36,37]:

(3) $$\begin{array}{c} V_{vdW}^{i}\left(r_{i}\right)=\varepsilon \left[\frac{6}{n(r_{i})-6}{\left(\frac{R_{m}}{r_{i}}\right)}^{n(r_{i})}-\frac{n(r_{i})}{n(r_{i})-6}{\left(\frac{R_{m}}{r_{i}}\right)}^{6}\right], \end{array}$$

where ε and $R_{m}$ are the well depth and its location for each interacting pair, respectively. This function gives a more realistic representation of both the repulsion and the long-range attraction than the classic Lennard–Jones potential [38]. The $n(r_{i})$ term is expressed as a function of both $r_{i}$ and $R_{m}$:

(4) $$n\left(r_{i}\right)=\beta +4.0{\left(\frac{r_{i}}{R_{m}}\right)}^{2},$$

where β is a parameter which depends on the hardness of the interacting particles. For all atom–atom pairs, β has been here fixed to 7, a value typical in neutral–neutral systems. Note that the adopted parameter values are connected to the effective atomic polarizability components within the molecules, whose combination is consistent with the global polarizability value. This provides the proper link between molecular deformation, due to bond stretching, and the modulation of van der Waals parameters.

In [18], the ε and $R_{m}$ parameters, referring to molecules kept at their intramolecular equilibrium distance, were fine-tuned by exploiting the comparison between experimental and calculated data of second virial coefficients as well as that between interaction potential model predictions and accurate ab initio electronic structure computations. The parameters values employed in this work are reported in Table 2.

The $V_{elect}$ term of Equation (1) is given as a sum of Coulomb potentials as follows:

(5) $$V_{elect}\left(R,\Theta_{a},\Theta_{b},\Phi \right)=\sum_{jk}\frac{q_{ja}q_{kb}}{r_{jk}},$$

with $q_{ja}$ and $q_{jb}$ being point charges (located on CO, monomer *a*, and N${}_{2}$, monomer *b*, respectively, and having values corresponding to calculated molecular dipole and quadrupoles) and $r_{jk}$ being the distance between them. For N${}_{2}$ charge values $q_{ja}$ we adopted those reported in ref. [39], while those for CO $q_{ja}$ are obtained as detailed in Appendix of [18].

The extrapolation of the rigid rotor PES, in order to include the dependence on flexible monomers, has also been described and tested in [18]. Specifically, this is implicitly introduced by considering the dependence of molecular polarizability and of a molecular electric dipole and electric quadrupole moments on the bond length of CO and N${}_{2}$. The reliability of the PES upon molecular elongation paves the way for dynamical calculations of collisions involving vibrationally excited N${}_{2}$ or CO.

### 2.2. Refinement of the PES over Experimental and Ab Initio Data

A preliminary calculation was carried out to investigate the ability of the original PES [18] to determine inelastic rate coefficients in a wide temperature range. Experimental data are available for V–V energy exchange processes of CO in nitrogen-containing mixtures: CO$\left(0\right)+N_{2}\left(1\right)\to$ CO$\left(1\right)+N_{2}\left(0\right)$ (hereafter indicated as (0,1) → (1,0) in short) and its inverse reaction. Calculations were therefore carried out by using the quasiclassical trajectory (QCT) approach through the VENUS code [40] and the mixed quantum-classical method described in Appendix A.

The collision dynamics for QCT calculations was treated in a classical mechanics framework. Accordingly, the cross section $\sigma_{v_{1}v_{2}\to v_{1}^{\prime }v_{2}^{\prime }}$ associated to the process CO($v_{1}$) + N${}_{2}$($v_{2}$) → CO($v_{1}^{\prime }$) + N${}_{2}$($v_{2}^{\prime }$) can be expressed as follows:

(6) $$\sigma_{v_{1}v_{2}\to v_{1}^{\prime }v_{2}^{\prime }}=2\pi \int_{0}^{b_{max}}P_{v_{1}v_{2}\to v_{1}^{\prime }v_{2}^{\prime }}\left(E,b\right)bdb$$

where *E* is the collision energy, and *b* is the impact parameter, running from 0 to the cutoff value $b_{max}$. $P_{v_{1}v_{2}\to v_{1}^{\prime }v_{2}^{\prime }}\left(E,b\right)$ is the probability for a single trajectory to lead to the above vibrational exchange. $P_{v_{1}v_{2}\to v_{1}^{\prime }v_{2}^{\prime }}$ can be estimated from a large number of trajectories, each starting from appropriate randomly selected initial conditions (except for *E*). Usually, the initial rotational angular momenta of the colliding molecules are randomly sampled from a Boltzmann distribution corresponding to a given rotational temperature. The momentum vectors were therefore randomly oriented and the initial vibrational energies of the molecules selected by matching the specified vibrational quantum numbers. The impact parameter *b* was also randomly selected in its range. Given a batch of trajectories, $P_{v_{1}v_{2}\to v_{1}^{\prime }v_{2}^{\prime }}$ is, therefore, the ratio between the number of trajectories leading to the above vibrational transition and the total number of trajectories. Such a selection scheme yields cross-sections not thermally averaged, but natively as a function of the collision energy *E*. Thermal averaging over translations at a given temperature T can be achieved by assigning different *E* values to the trajectories, as obtained from a sampling of the Boltzmann distribution at temperature T. The final cross-sections are thus specific for the vibrational states, but thermally averaged, also over rotations at the given rotational temperature, which is assumed to be equal to the translational one. A data-binning procedure is adopted to assign the final vibrational quantum numbers to the collision trajectories. Thermal state-specific rate coefficients for the above transition (e.g., vibrational) can be expressed as follows:

(7) $$k\left(T\right)={\left(\frac{8k_{B}T}{\pi \mu }\right)}^{1/2}\sigma \left(T\right).$$

The entire set of rate coefficients presented in the next section has been obtained from computational batches amounting to approximately 50 × 10${}^{6}$ collision trajectories, at each temperature.

QC calculations were carried out by running trajectories at 45 initial values of total classical energy comprised between 35 ${\mathrm{cm}}^{-1}$ and 80,000 ${\mathrm{cm}}^{-1}$, with a more frequent sampling directed towards lower energies. For each classical energy value, 2000 trajectories were considered, which should ensure an accuracy for rate coefficients of ca. 15% at low and ca. 10% at high temperatures. An initial separation of the diatoms equals to 15 Å for V–V energy transfer and an impact parameter randomly chosen between 0 and 9 Å is employed. For the above processes, 36 initial vibrational states were considered in the set of coupled time-dependent quantum equations. Note that a smaller number of trajectories is needed in QC than in the QCT method to reach convergence, as a result of having two quantum degrees of freedom. Therefore, the longer computational time associated with the calculation of one *quantum* trajectory is balanced by a limited number of trajectories to be computed.

**Figure 2:**
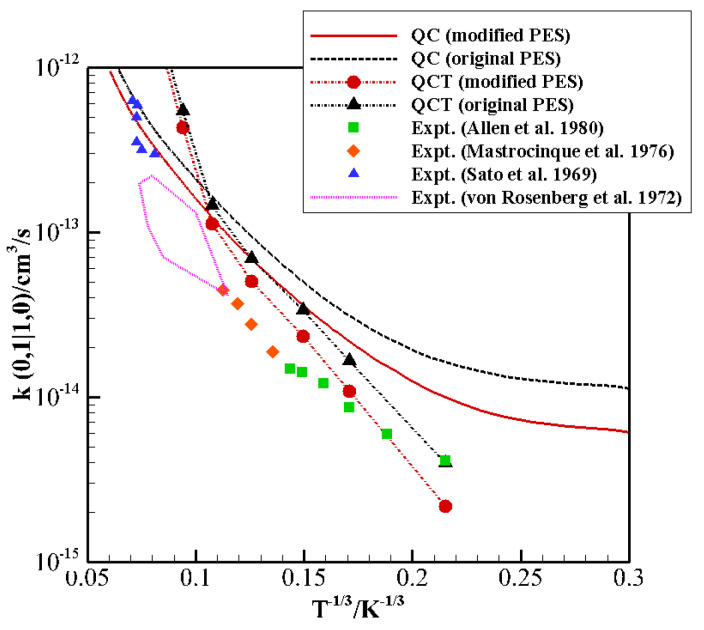
Landau–Teller plot of the rate coefficients for the transition (0,1) → (1,0) calculated by the QC method on the original PES (dash black line) and on the modified PES (full red line), and by the QCT method on the original PES (dash-dot black line with triangle symbols) and on the modified PES (dash-dot red line with circle symbols). Experimental data of (green squares) [41], (orange diamonds) [42], (blue triangles) [43], and (solid pink line) [44] are also reported.

As shown in Figure 2, QCT results on the original PES for the exothermic (0,1) → (1,0) collision give an excellent agreement with the experimental data at the lowest temperature. On the other hand, as temperature grows, QCT rates become larger and they rapidly increase, with an abrupt change of slope. QC results, on the contrary, overestimate the experimental values in the whole temperature range, with the largest discrepancy, of a factor of 3 at most, at the lowest investigated temperature [41,42]. The experimental slope is however basically reproduced.

**Figure 3:**
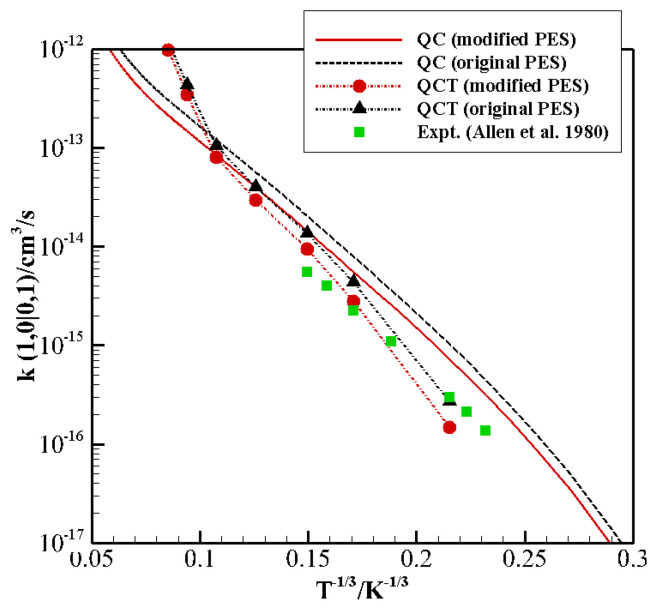
Landau–Teller plot of rate coefficients for the transition (1,0) → (0,1) calculated by the QC method on the original PES (dash black line) and on the modified PES (full red line), and by the QCT method on the original PES (dash-dot black line with triangle symbols) and on the modified PES (dash-dot red line with circle symbols). Experimental data of ref. [41] (green squares) are also reported.

For the endothermic (1,0) → (0,1) quasi-resonant exchange, experimental data is available in the low-temperature regime (80–300 K) [41]. The calculated rate coefficients (Figure 3) show the same behavior as for the exothermic transition (0,1) → (1,0), with QCT agreeing well with the experimental data at low and intermediate temperature, but again showing a sudden increase at high T, and QC results overestimating the data but reproducing the correct experimental trend in the whole temperature range.

This behavior suggests that a refinement of the original PES parameters might lead to an improved quantitative agreement with experimental data. This is particularly important here, since the main motivation of the work is the calculation of a large accurate database of inelastic rate coefficients. The nature of the formulation of the present PES is particularly helpful in this sense, because it allows the investigation of the contributions to the collision coming from different interaction regions or different configurations.

**Figure 4:**
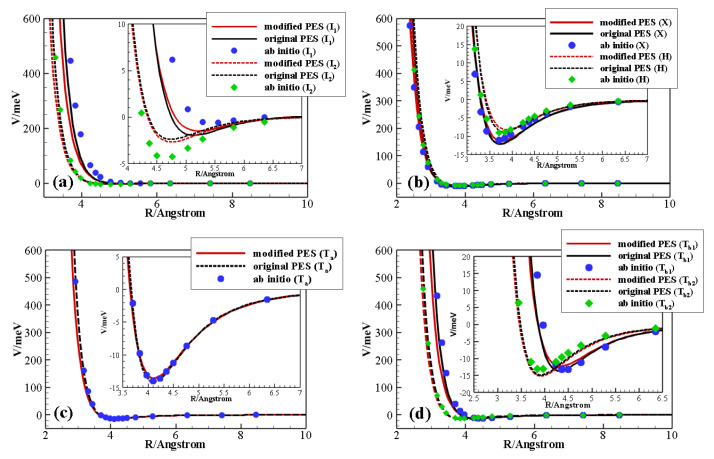
Behavior of different potential energy surfaces as a function of the diatomic interaction distance. Selected configurations, at the equilibrium intramolecular diatomic distance of both monomers, are considered: I${}_{1}$ and I${}_{2}$ (panel **a**), X and H (panel **b**), T${}_{a1}$ (panel **c**), and T${}_{b1}$ and T${}_{b2}$ (panel **d**).

To such purpose, significant cuts of the original PES were compared to the corresponding supermolecular ab initio energies. The latter were calculated at the CCSD(T) level of theory, using Dunning’s aug-cc-pVQZ basis set [45] and the bond function set [3s3p2d1f] developed by Tao [46] and placed on the midpoint of the intermolecular distance *R*. The Molpro code [47] was employed for ab initio calculations. The interaction energies were corrected using the counterpoise method [48] in order to remove the basis set superposition error. Such cuts, representing the potential energy as a function of the centers of mass distance *R* of the diatoms at their equilibrium geometry, are reported in Figure 4 for limiting configurations (specifically, H: ($\Theta_{a},\Theta_{b},\Phi$) = ($90^{\circ }$, $90^{\circ }$, $0^{\circ }$), X: ($\Theta_{a},\Theta_{b},\Phi$) = ($90^{\circ }$, $90^{\circ }$, $90^{\circ }$), $T_{a}$: ($\Theta_{a},\Theta_{b},\Phi$) = ($90^{\circ }$, $0^{\circ }$, $0^{\circ }$), $T_{b1}$: ($\Theta_{a},\Theta_{b},\Phi$) = ($0^{\circ }$, $90^{\circ }$, $0^{\circ }$), $T_{b2}$: ($\Theta_{a},\Theta_{b},\Phi$) = ($180^{\circ }$, $90^{\circ }$, $0^{\circ }$), $I_{1}$:($\Theta_{a},\Theta_{b},\Phi$) = ($0^{\circ }$, $0^{\circ }$, $0^{\circ }$) and $I_{2}$: ($\Theta_{a},\Theta_{b},\Phi$) = ($180^{\circ }$, $0^{\circ }$, $0^{\circ }$)).

The comparison shows that this PES gives an excellent description at long range and very short range in all cases, and the interaction well shows a very good agreement for the X, H, T${}_{a1}$, T${}_{b1}$, and T${}_{b2}$ configurations. However, there are non-negligible discrepancies for the well depth and location for the collinear I${}_{1}$ and I${}_{2}$ configurations, which are considered to be the most effective for the exchange of vibrational quanta of energy. QC results in Figure 2 and Figure 3 show a general tendency to overestimate experimental values, a manifestation of the necessity of improving the description of the interaction in the potential wells and at short range. We thus focused on the refinement of the parameters affecting these regions in the collinear configurations.

The values of ε corresponding to the C-N and O-N pairs were decreased by 16.5% and increased by 7.8%, respectively (see Table 2). Moreover, in order to have a gentler short-range repulsive wall, β was decreased from 8 to 7. There are only slight changes in the other configurations, whose energy values as a function of *R* were already very close to the ab initio points. The modified PES indeed leaves the description of all configurations, but the collinear ones, practically unchanged (see Figure 4); there is however a significant improvement for the I${}_{1}$ geometry (both well depth and position) and a slight one for I${}_{2}$. We recall that, in the present approach, parameters are not allowed to be freely varied: their values have to maintain a physical meaning for the PES to provide a good description in all regions. In particular, an isotropic dispersion coefficient $C_{6}$ can be extracted from the global attraction in the asymptotic region. Its value, $C_{6}$ = 40.43 eV Å${}^{2}$, is in good agreement (within 10%) with the value $C_{6}$ = 44.97 eV Å${}^{2}$ reported in literature [49].

The modified PES was used to calculate again the rate coefficients of V–V resonant exchange CO$\left(0\right)+N_{2}\left(1\right)\to$ CO$\left(1\right)+N_{2}\left(0\right)$ and its inverse transition (Figure 2 and Figure 3, respectively). QCT rate coefficients at low temperature now provide an excellent agreement with the experimental data, though the qualitative slope remains different from the experiment, with large differences at high temperature. The QC rate coefficients on the modified PES show an overall average decrease and thus present a better agreement with experimental data both at high and low temperatures. The still-existing differences (about a factor of 2) at low T could be due to the accuracy of low-temperature experiments or to the neglecting of a proper quantum treatment for rotations which might play a role in the vibrational energy exchange process at very low collision energies.

The modified PES was also tested on other experimental data corresponding to different physical properties. In [18], it was shown that the original PES provided good results for the calculation of the second virial coefficient, B(T), values, including first quantum correction $B_{ql}\left(T\right)$ to the classical estimate $B_{cl}\left(T\right)$ [50] ($B\left(T\right)=B_{q1}\left(T\right)+B_{c1}\left(T\right)$). The same calculation carried out on the modified PES leads to similarly good results with some slight improvement in the comparison with the experimental measurements [51,52] for temperatures below 305 K (Table 3). Note that the Boyle temperature, $T_{B}$ ($B(T_{B})$ = 0), which depends on the critical balance of attraction and repulsion, is predicted to be around 330 K and is consistent with experimental determinations (320 $<T_{B}<$ 340 K). Thus, correct behavior of the modified PES in the well region leads to a sensible improvement in reproducing inelastic rates and second virial coefficients in the whole temperature range.

**Figure 5:**
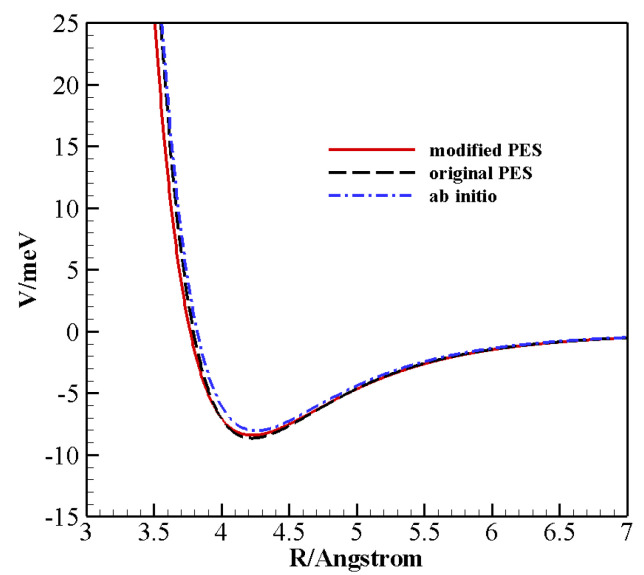
Spherical average of different potential energy surfaces as a function of the diatomic interaction distance. The ab initio results are those from Ref. [25].

Figure 5 shows the spherical average of the original and modified PES together with that obtained from ab initio data [25] as a function of *R*. This is a valuable test because the average attraction at long range can be directly connected to the average component of the total scattering cross-sections, whereas quantum interference effects arise from features of the interaction in the potential well region. The modified PES shows an isotropic component consistent with that of the original one and ab initio data [25]. A non-negligible discrepancy with respect to the ab initio data only occurs in the very repulsive region (in the first repulsive wall the behavior of the original and modified PESs is quite similar). That region of the potential is only accessible for very energetic collisions and is likely to have consequences at very high temperatures. At the highest temperature investigated here, the rate coefficients computed on the two PESs present small differences, and experimental data fall in between the two calculations (Figure 2). At all other temperatures, the modified PES gives values closer to the experiment. Furthermore, we would like to stress that such a softer repulsive wall leads to a slight improvement of the comparison with experimental second virial coefficients (Table 3), a property that is most sensitive to the isotropic component of the global interaction.

## 3. Results and Discussion

As mentioned in Section 1, an existing database of V–V rate coefficients, obtained by using a QC method, is available [11]. Here we extend the calculations to include a larger number of V–V transitions and a wider temperature range (20–7000 K). Furthermore, we calculated V–T/R single and multiquantum energy exchange rate coefficients, whose determination is a computationally demanding task, because they need larger initial diatomic separation distances and thus much longer simulation times. For this reason, to the best of our knowledge, this is the first V–T/R rates database for CO-N${}_{2}$ collisions covering a wealth of excited states and temperatures. Because of the high dissociation energies of the two molecules, reactive channels for these collisions are likely to play a role even at the highest temperature investigated here. Dissociation rate coefficients calculated for the similar N${}_{2}$-N${}_{2}$ system [53] show that reactivity starts to be significant for temperatures much above 8000 K.

V–V rate coefficients involving highly excited vibrational states were calculated by coupling 121 initial vibrational states while the other settings are the same of the (0,1) → (1,0) calculations (i.e. by starting from an initial diatoms separation distance equal to 15 Å and 45 different values of total classical energies, comprised between 35 cm${}^{-1}$ and 80,000 cm${}^{-1}$). For the calculation of V–T/R rate coefficients, 81 vibrational states were coupled for 10≤v≤30 and 121 for v>30, the larger number being needed for higher *v* because of the close spacing between the levels. In this case, an initial diatoms separation distance equal to 80 Å has been considered, needed for V–T/R processes to avoid artificial contributions from the long-range part of the multipole moments.

The dependence of the present PES on the intramolecular distance has been tested in ref. [18]: molecular polarizabilities of both molecules, N${}_{2}$ quadrupole moment and CO permanent dipole moment depend on the corresponding bond lengths. Its accuracy might, however, slightly decrease for very elongated monomers. Furthermore, Morse potential and Morse wavefunctions also lose accuracy when describing very high vibrational states. Rate coefficients for processes involving vibrational states with v≥35 might therefore present an overall uncertainty larger than 20%. However, we believe them to be sufficiently reliable (more than those available by extrapolation or first-order treatments) for such processes and to provide at least the correct qualitative trend in their variation.

A comparison between some exemplary symmetric single quantum and asymmetric multiquantum near-resonant V–V rate coefficients calculated on the present PES and those of Ref. [11] can be found in Table 4. The present values are a factor two larger ca., the discrepancy growing larger with temperature, which could be connected to the more repulsive short-range character of the present PES.

In many applications, nitrogen molecules only populate the lower vibrational levels because of a fast energy transfer from N${}_{2}$ to CO molecules [54]. Therefore, the rate coefficients for single quantum V–V processes CO$\left(v\right)+N_{2}\left(1\right)\to$ CO$\left(v+1\right)+N_{2}\left(0\right)$ (Table 5) and near-resonant asymmetric V–V processes CO$\left(v-2\right)+N_{2}\left(1\right)\to$ CO$\left(v\right)+N_{2}\left(0\right)$ (Table 6) and CO$\left(v-2\right)+N_{2}\left(2\right)\to$ CO$\left(v\right)+N_{2}\left(1\right)$ (Table 7) are of particular importance. The rate coefficients for processes CO$\left(1\right)+N_{2}\left(v\right)\to$ CO$\left(0\right)+N_{2}\left(v+1\right)$ are also reported in Table 5.

**Figure 6:**
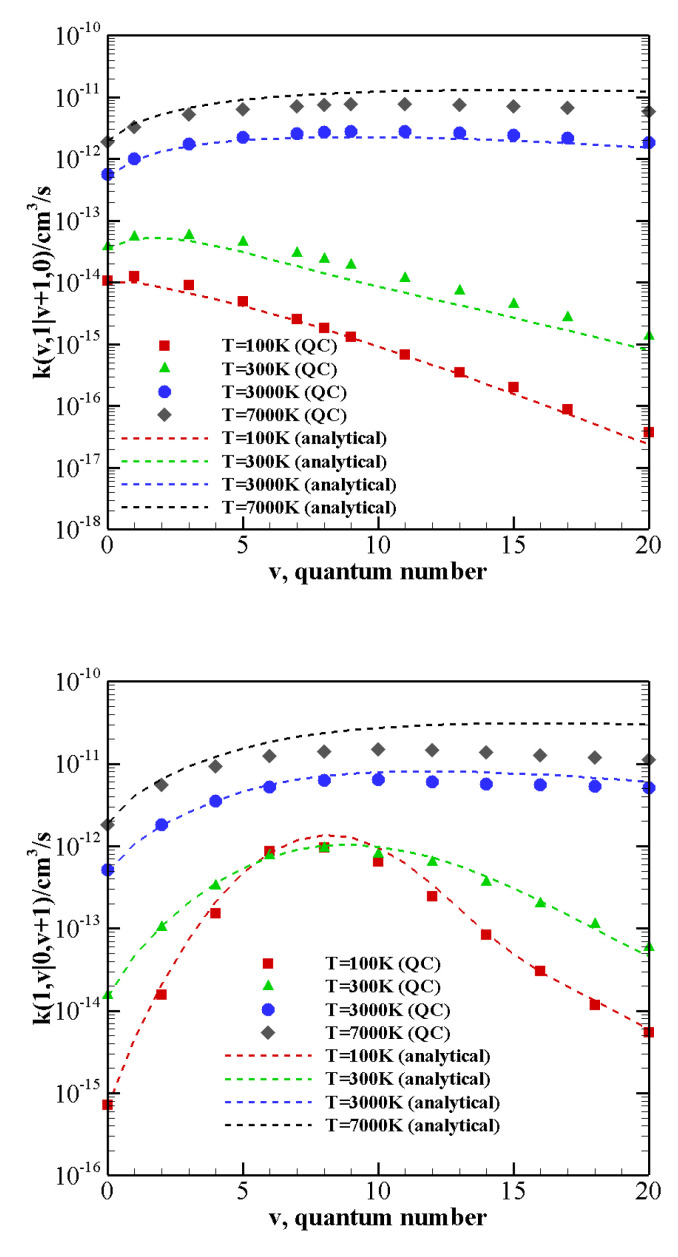
Rate coefficients for the V–V transitions as a function of the vibrational quantum number *v* at different temperature values. (**upper panel**) CO$\left(v\right)+N_{2}\left(1\right)\to$ CO$\left(v+1\right)+N_{2}\left(0\right)$; (**lower panel**) CO$\left(1\right)+N_{2}\left(v\right)\to$ CO$\left(0\right)+N_{2}\left(v+1\right)$. Dash lines correspond to the analytical rate coefficients obtained by the modified SSH theory (Appendix B).

Figure 6 shows the QC calculated V–V rate coefficients (symbols) for the exothermic CO$\left(v\right)+N_{2}\left(1\right)\to$ CO$\left(v+1\right)+N_{2}\left(0\right)$ processes and for CO$\left(1\right)+N_{2}\left(v\right)\to$ CO$\left(0\right)+N_{2}\left(v+1\right)$ processes as a function of the vibrational quantum number *v* at 100 K, 300 K, 3000 K, and 7000 K. The analytical approximation for the rate coefficients obtained by the modified SSH theory using the present QC results (see Appendix B) is also reported in the figure (dashed lines).

At high temperatures the quenching rate of N${}_{2}\left(1\right)$ (or CO$\left(1\right)$) stimulated by CO$\left(v\right)$ (or N${}_{2}\left(v\right)$) depends little on *v*. Although the reaction energy ΔE increases with the vibrational quantum number, the probability of quenching the first excited state remains not negligible even for the highest vibrationally excited states investigated here. At low temperatures, quasi resonant processes CO$\left(1\right)+N_{2}\left(v\right)\to$ CO$\left(0\right)+N_{2}\left(v+1\right)$, with v≈8, are the most active to promote quenching and show orders of magnitude differences with the other processes.

The comparison between the QC and analytical rates is quite good at low temperature and for small energy mismatches. At high temperatures, however, the difference grows and the analytical formulation, consistently with the known drawbacks of the SSH theory, fails to correctly reproduce rate coefficients.

**Figure 7:**
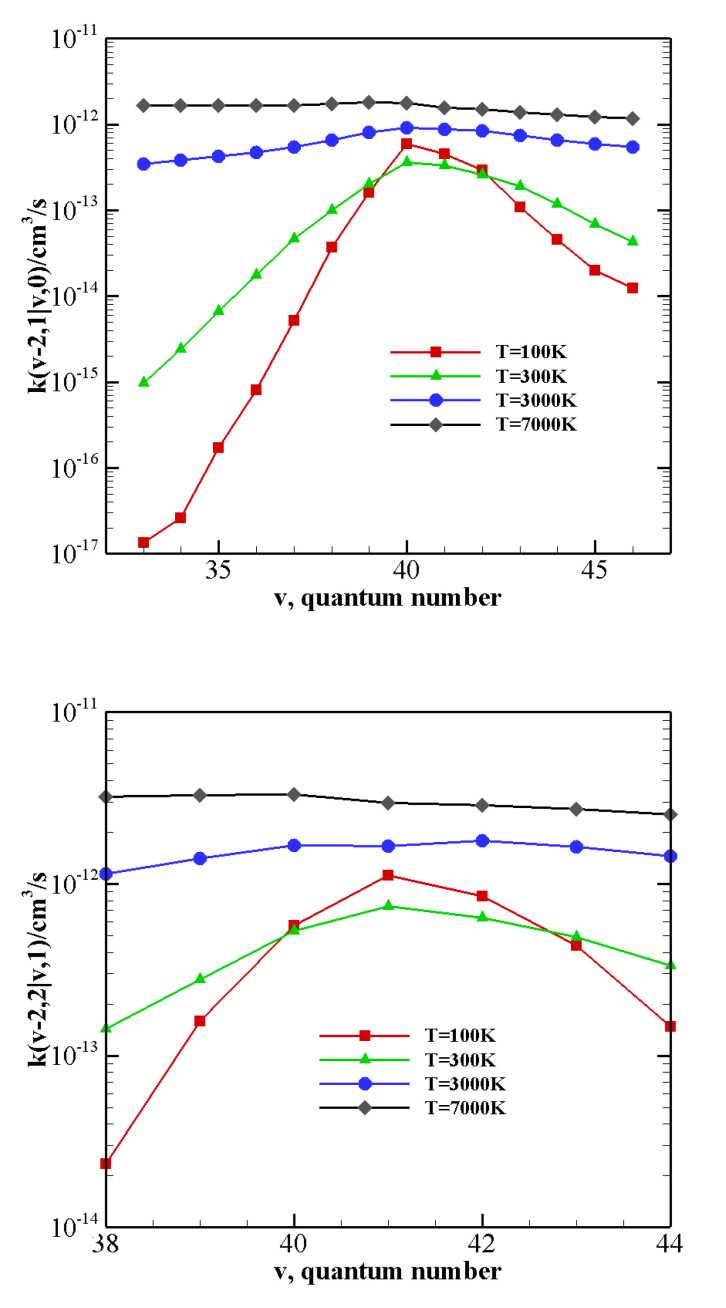
V–V rate coefficients for the near-resonant asymmetric transitions as a function of the vibrational quantum number *v* at different temperature values. (**upper panel**) CO$\left(v-2\right)+N_{2}\left(1\right)\to$ CO$\left(v\right)+N_{2}\left(0\right)$; (**lower panel**) CO$\left(v-2\right)+N_{2}\left(2\right)\to$ CO$\left(v\right)+N_{2}\left(1\right)$.

The above-described behavior both at high and low temperature is enhanced by the vibrational anharmonicity for the near-resonant asymmetric transitions occurring in N${}_{2}$-CO collisions with vibrationally excited CO, i.e., CO$\left(v-2\right)+N_{2}\left(1\right)\to$ CO$\left(v\right)+N_{2}\left(0\right)$ and CO$\left(v-2\right)+N_{2}\left(2\right)\to$ CO$\left(v\right)+N_{2}\left(1\right)$. The corresponding rate coefficients are reported in Figure 7 as a function of the vibrational quantum number *v* at 100 K, 300 K, 3000 K, and 7000 K. At high temperature, rate coefficients for such transitions are practically independent on the CO vibrational quantum number and are therefore expected to play an important role in the vibrational kinetics of highly excited CO molecules [55]. At low temperature rates, coefficients strongly grow as the transition becomes more resonant. For nearly resonant transitions in the low-temperature regime a marked anti-Arrhenius behavior, i.e., rate coefficients getting smaller with temperature, is found, as reported in Figure S1 in the SI for the CO$\left(38\right)+N_{2}\left(1\right)\to$ CO$\left(40\right)+N_{2}\left(0\right)$ transition.

V–T/R rate coefficients for processes where vibrational relaxation occurs due to the collision between N${}_{2}$ (CO) in its vibrational ground state and a vibrationally excited CO (N${}_{2}$) molecule, with the loss of a single quantum of vibrational energy are collected in Table 8 (Table 9). V–T/R rate coefficients corresponding to the loss of two or three vibrational quanta are reported in Table 10 and Table 11. They show that, though the rate of V–T/R processes is very small at low temperature (being generally some orders of magnitude smaller than V–V processes for the same initial vibrational states, see also the following), their efficiency rapidly grows by increasing the temperature. Indeed, V–T/R rates become comparable to V–V ones at high temperatures, and in some cases, they even correspond to the most efficient energy transfer events. Table 10 and Table 11 show that even multiquantum V–T/R transitions at high temperature are not negligible, especially for highly excited molecules.

**Figure 8:**
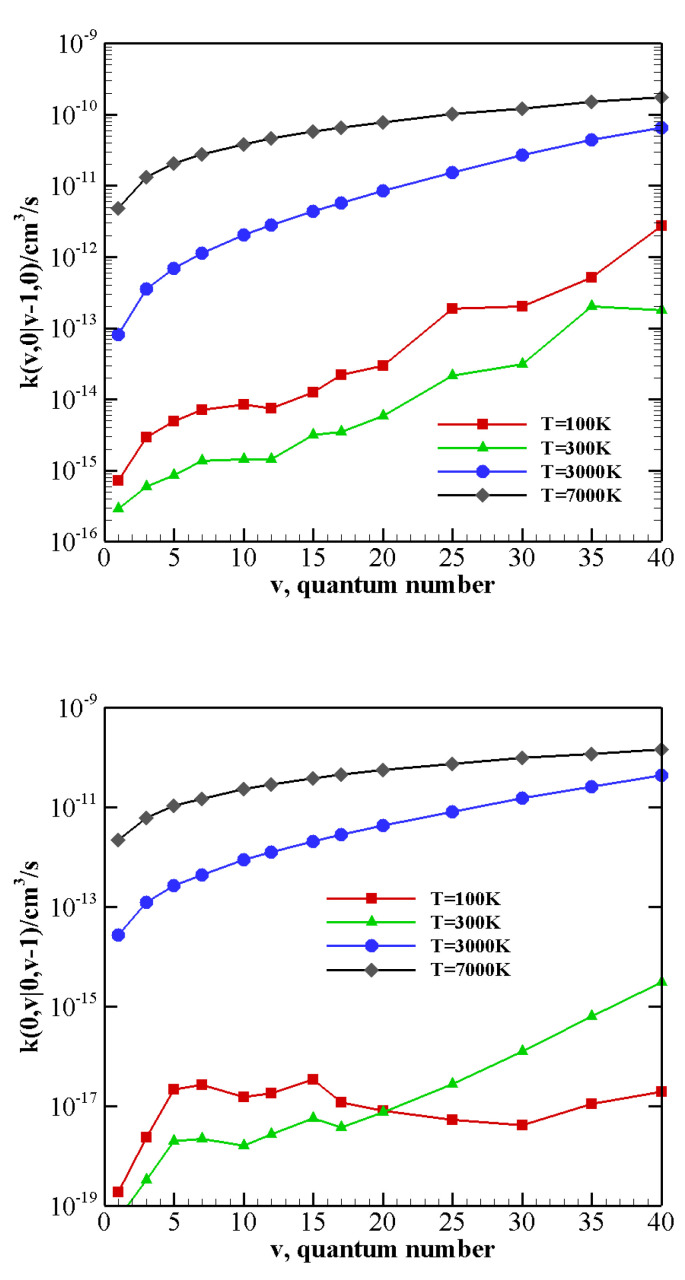
Single quantum V–T/R rate coefficients as a function of the vibrational quantum number *v* at different temperature values for transitions (**upper panel**) CO$\left(v\right)+N_{2}\left(0\right)\to$ CO$\left(v-1\right)+N_{2}\left(0\right)$; (**lower panel**) CO$\left(0\right)+N_{2}\left(v\right)\to$ CO$\left(0\right)+N_{2}\left(v-1\right)$.

**Figure 9:**
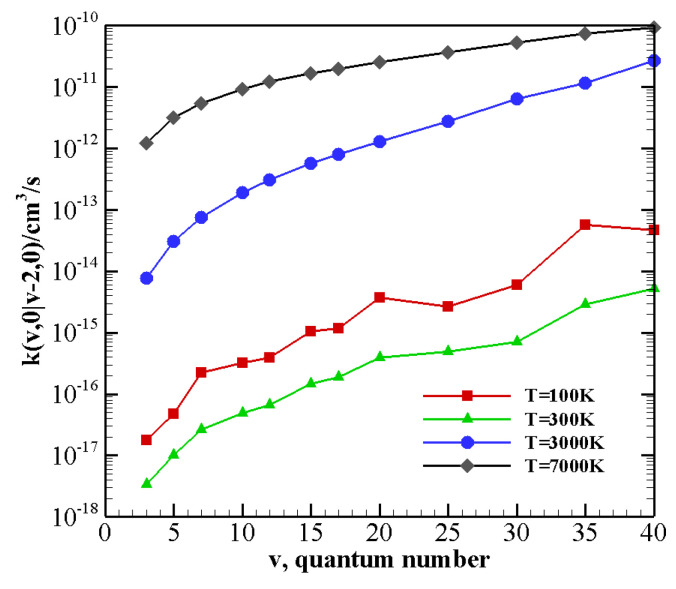

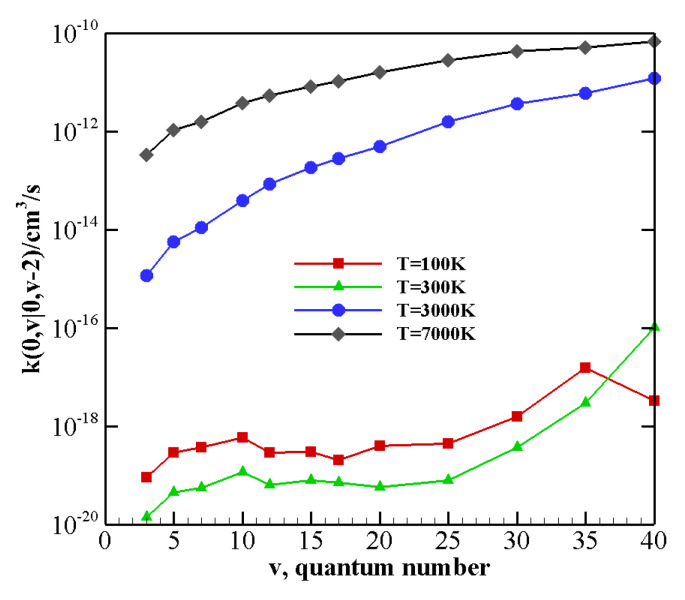
Multiquantum V–T/R rate coefficients as a function of the vibrational quantum number *v* at different temperature values for transitions (**upper panel**) CO$\left(v\right)+N_{2}\left(0\right)\to$ CO$\left(v-2\right)+N_{2}\left(0\right)$; (**lower panel**) CO$\left(0\right)+N_{2}\left(v\right)\to$ CO$\left(0\right)+N_{2}\left(v-2\right)$.

**Figure 10:**
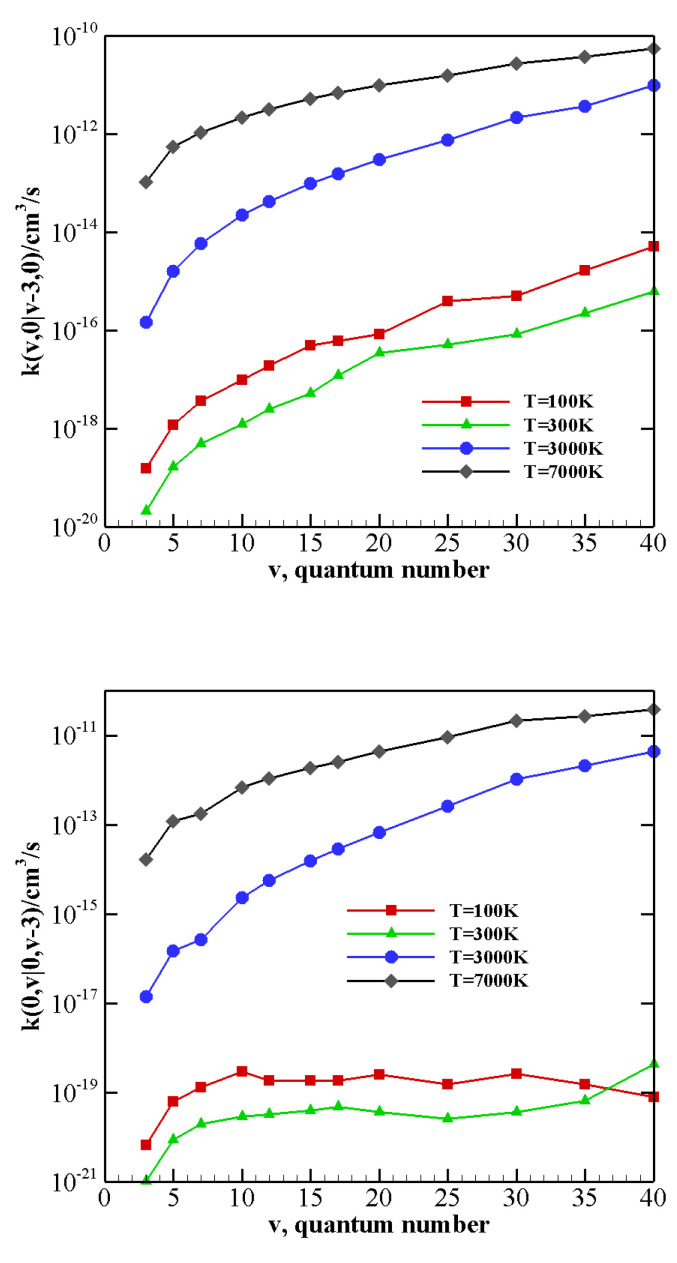
Multiquantum V–T/R rate coefficients as a function of the vibrational quantum number *v* at different temperature values for transitions (**upper panel**) CO$\left(v\right)+N_{2}\left(0\right)\to$ CO$\left(v-3\right)+N_{2}\left(0\right)$; (**lower panel**) CO$\left(0\right)+N_{2}\left(v\right)\to$ CO$\left(0\right)+N_{2}\left(v-3\right)$.

The behavior of rate coefficients for V–T/R processes CO(v) + N${}_{2}$(0)→ CO(v−w) + N${}_{2}$(0) and CO(0) + N${}_{2}$(*v*)→ CO(0)+N${}_{2}$(v−w), as a function of the initial quantum number *v* at T = 100, 300, 3000, and 7000 K is reported in Figure 8, Figure 9 and Figure 10 for w=1,2,3, respectively. All the figures show a pronounced increase of vibrational relaxation when increasing the initial *v* value of either N${}_{2}\left(v\right)$ or CO(v) molecule at high temperatures. At low temperatures, V–T/R vibrational relaxation of CO(v) is more efficient than N${}_{2}\left(v\right)$, as a result of the closer vibrational spacing in CO. It is interesting to note that an anti-Arrhenius behavior at low temperatures characterizes the vibrational relaxation of CO(*v*) for all *v* values investigated here, whereas for the quenching of N${}_{2}$(*v*) the standard Arrhenius trend is followed when *v* > 20.

The multiquantum V–T/R rate coefficients for CO(v) + N${}_{2}$(0)→ CO(v−w) + N${}_{2}$(0) show a very similar qualitative behavior to that of single quantum rates, with correspondingly lower values. For CO(0) + N${}_{2}$(*v*)→ CO(0) + N${}_{2}$(v−w) processes, at low temperatures, the rate coefficients for the loss of vibrational quanta are practically constant until a sufficiently high value of *v* is reached. Moreover, with respect to the corresponding single quantum rates, the Arrhenius behavior at low temperatures is restored at higher *v* values.

It is worth noting that, at low temperatures, many V–T/R rate coefficients are very small and close to the numerical accuracy of the present method, so that such values may not be so accurate as larger ones. However, we believe that they give a correct qualitative indication of the general trend of these processes.

It is important to compare the relative efficiency of V–V and V–T/R processes in different temperature regimes. This is done in Table 12 where single quantum V–V and V–T/R rate coefficients are reported. V–V processes largely dominate at low temperatures and, though V–T/R rates enhancement with temperature is much more rapid, they remain predominant at the highest temperature investigated here, when V–T/R coefficients are barely the same order of magnitude.

**Figure 11:**
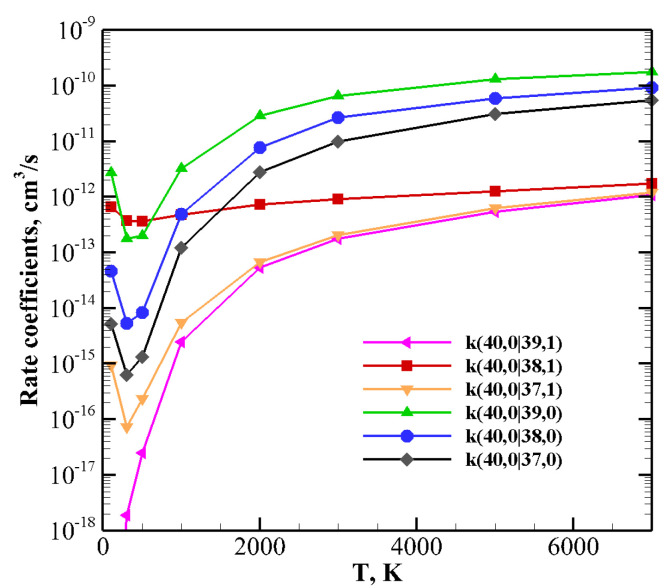
V–T/R and V–V rate coefficients for selected CO$\left(40\right)+N_{2}\left(0\right)$ processes as a function of temperature.

**Figure 12:**
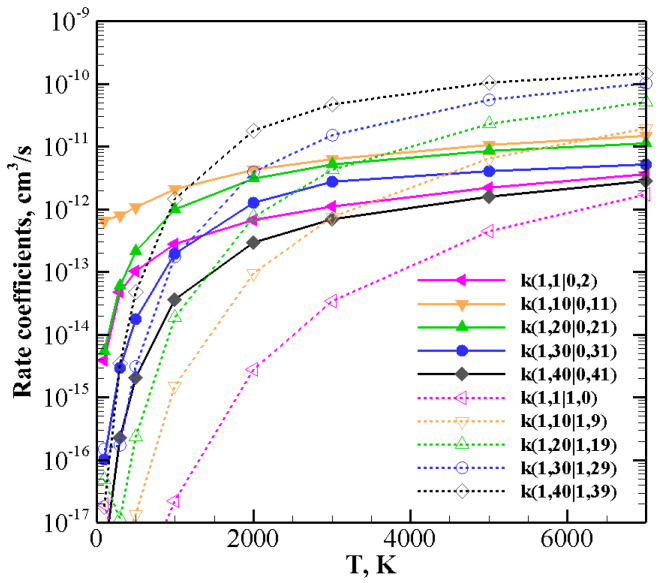
Rate coefficients for V–V (solid lines) and V–T/R (dashed lines) exothermic processes for CO(1) + N${}_{2}$(*v*) collisions.

**Figure 13:**
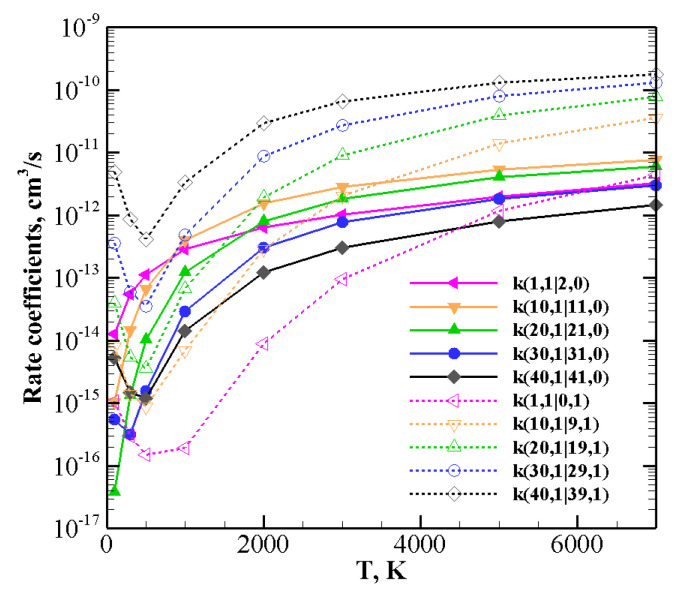
Rate coefficients for V–V (solid lines) and V–T/R (dashed lines) exothermic processes for CO(*v*) + N${}_{2}$(1) collisions.

Things change when one of the molecules is highly vibrationally excited and the other one is in the ground (Figure 11) or in the lowest vibrational excited state v=1 (Figure 12 and Figure 13).

For collision between CO(1) and highly excited N${}_{2}$(*v*), rate coefficients for V–V (Table 5) and V–T/R (Table 13) exothermic processes are reported in Figure 12: for the lowest *v* value (v=1) V–V processes always predominate. However, as *v* grows, V–T/R processes are faster at high temperatures and they become the most probable events for the highest value investigated here, v=40, in the whole temperature range. When collisions between N${}_{2}$(1) and highly excited CO(*v*) (Table 5 and Table 14), corresponding to exothermic processes, are considered (Figure 13), the above behavior is enhanced. V–T/R always predominate at the highest temperature, and they often do in the low-temperature regime as well. In fact, the anti-Arrhenius behavior occurring at the lowest temperature tends to favor V–T/R processes over V–V ones.

The same indication is found in Figure 11, which reports the rate coefficients of the collision between a highly vibrationally excited CO$\left(40\right)$ molecule and N${}_{2}$ at its ground state. Here both endothermic and exothermic processes are included: V–T/R relaxation tends to be predominant in the whole temperature range, with the exception of the quasi-resonant CO(40) + N${}_{2}$(0) → CO(38) + N${}_{2}$(1) process, which, at low temperatures, is most efficient.

These results suggest that, in the high-temperature range and/or when highly excited states are considered, V–T/R relaxation processes are competitive or more effective than V–V ones. These conditions are those commonly found in plasmas, hypersonic flows, and other situations of interest in aerospace science, therefore V–T/R databases are useful not only for numerical simulation of the above scenarios but also a valuable source for comparison and interpretation of laboratory-based experiments.

## 4. Conclusions

A recent analytical PES for non-reactive collisions between CO and N${}_{2}$ was critically improved to be used for the construction of a large reliable database of V–V and V–T/R rate coefficients. This is possible thanks to the ILJ formulation of the potential, which gives the opportunity of modulating physically meaningful parameters by looking at the stereodynamical behavior of the system and by checking and analyzing the performance of the PES against high-level ab initio and experimental data.

QCT and QC rate coefficients for (1,0) → (0,1) and (0,1) → (1,0) transitions were determined, for which experimental data are available, showing a better agreement than the original PES, particularly at low temperature. QCT calculations show an excellent agreement at low temperature, but fail to reproduce the correct trend in the whole temperature range, whereas QC ones provide the correct slope of the Landau–Teller plots and a more accurate overall behavior. In fact, the new potential improves the description of the inelastic scattering dynamics also in the high temperature regime, most important for the modeling of hypersonic flows and aerospace applications.

We find that, in the above-mentioned high-temperature conditions, V–T/R coefficients, whose calculation represents an additional challenge, due to the required computational time, are generally comparable or even larger to V–V ones. This makes the determination of these quantities an important step towards the accurate modeling of combustion processes, satellite or spacecraft re-entry conditions, etc. The present database is at the date the first one containing a large number of V–T/R rate coefficients.

We would like to conclude by pointing out that the present investigation is one of a series where a systematic approach is used to build PESs in an internally coherent fashion, i.e., by using a physically meaningful analytical formulation of the potential performing well in wide temperature ranges, tested against ab initio calculations and available experimental properties of different kinds. Such potentials are then used for the determination of large bodies of inelastic scattering cross-sections, involving vibrational energy transfer. Work is still in progress to extend such methodology to other diatom–diatom and diatom–atom systems.

## Figures and Tables

**Table 1:** Molecular constants for CO and N${}_{2}$ [34].

	CO	N${}_{2}$
$\omega_{e}$ (${\mathrm{cm}}^{-1}$)	2169.81	2359.60
$x_{e}$	0.006125	0.006126
$y_{e}$	0.0000048	0.0000032
$r_{e}$ (Å)	1.128	1.098
$\beta_{e}$ (Å${}^{-1}$)	2.298	2.689
$D_{e}$ (eV)	11.07	9.905

**Table 2:** Parameters for the CO-N${}_{2}$ intermolecular potentials. $R_{m}$ (Å) and ε (meV) values define the vdW components in the corresponding atom–atom pair.

Atom–Atom	Original PES [18]			Modified PES		
	$R_{m}$	ε	β	$R_{m}$	ε	β
C-N	3.855	3.788	8.0	3.920	3.163	7.0
O-N	3.666	3.338	8.0	3.666	3.600	7.0

**Table 3:** Calculated and experimental second virial coefficient B(T) in cm${}^{3}$/mol as a function of temperature.

*T* (K)	Original PES [18]	Modified PES	Expt. [51]	Expt. [52]
200.0	−38.046	−37.714		
220.0	−28.546	−28.435		
240.0	−20.928	−20.990		
260.0	−14.694	−14.897		
270.0	−11.987	−12.250		
273.1	−11.183	−11.464	−12.4	−12.0
280.0	−9.508	−9.827		
290.0	−7.230	−7.601		
293.1	−6.551	−6.937	−7.0	
300.0	−5.132	−5.550		
303.1	−4.505	−4.937		−6.3
310.0	−3.193	−3.655		
313.1	−2.612	−3.087	−1.8	
318.1	−1.718	−2.214		−3.8
325.0	−0.547	−1.069		
333.1	0.774	0.221	2.9	−1.2
348.1	3.014	2.409		0.7
400.0	9.256	8.501		
500.0	17.097	16.138		
600.0	21.908	20.808		

**Table 4:** Comparison of V–V rate coefficients (in cm${}^{3}$/s) calculated with the present PES and those of Ref. [11] for CO$\left(v_{1}\right)+N_{2}\left(v_{2}\right)\to$ CO$\left(v_{1}^{\prime }\right)+N_{2}\left(v_{2}^{\prime }\right)$ transitions.

	$v_{1},v_{2}$	→	$v_{1}^{\prime },v_{2}^{\prime }$		200 K	500 K	800 K	1100 K	1400 K	1700 K	2000 K	2900 K
present	10, 11	→	9, 12		2.98E-12	7.03E-12	1.13E-11	1.59E-11	2.07E-11	2.51E-11	2.93E-11	4.09E-11
Ref. [11]	10, 11	→	9, 12		1.88E-12	3.73E-12	4.95E-12	6.24E-12	7.64E-12	9.09E-12	1.05E-11	1.46E-11
present	40, 0	→	38, 1		4.27E-13	3.68E-13	4.27E-13	5.07E-13	5.87E-13	6.60E-13	7.25E-13	8.91E-13
Ref. [11]	40, 0	→	38, 1		2.60E-13	1.84E-13	1.91E-13	2.15E-13	2.41E-13	2.65E-13	2.85E-13	3.34E-13

**Table 5:** Single quantum V–V rate coefficients (in cm${}^{3}$/s) for the transition CO$\left(v_{1}\right)+N_{2}\left(v_{2}\right)\to$ CO$\left(v_{1}^{\prime }\right)+N_{2}\left(v_{2}^{\prime }\right)+\Delta E$.

				$k_{v_{1},v_{2}\to v_{1}^{\prime },v_{2}^{\prime }}$							
$v_{1},v_{2}$	→	$v_{1}^{\prime },v_{2}^{\prime }$	$\Delta E\left({\mathrm{cm}}^{-1}\right)$	100 K	300 K	500 K	1000 K	2000 K	3000 K	5000 K	7000 K
0, 1	→	1, 0	187.46	1.06E-14	3.75E-14	7.02E-14	1.62E-13	3.54E-13	5.60E-13	1.11E-12	1.89E-12
1, 1	→	2, 0	213.94	1.27E-14	5.49E-14	1.14E-13	2.87E-13	6.42E-13	1.02E-12	2.00E-12	3.29E-12
3, 1	→	4, 0	266.73	8.95E-15	5.66E-14	1.43E-13	4.50E-13	1.08E-12	1.73E-12	3.33E-12	5.21E-12
5, 1	→	6, 0	319.27	4.96E-15	4.43E-14	1.32E-13	5.05E-13	1.36E-12	2.24E-12	4.25E-12	6.41E-12
7, 1	→	8, 0	371.55	2.56E-15	2.98E-14	1.06E-13	4.87E-13	1.51E-12	2.59E-12	4.89E-12	7.21E-12
8, 1	→	9, 0	397.60	1.83E-15	2.37E-14	9.21E-14	4.63E-13	1.54E-12	2.71E-12	5.13E-12	7.48E-12
9, 1	→	10, 0	423.59	1.31E-15	1.88E-14	7.87E-14	4.33E-13	1.54E-12	2.78E-12	5.29E-12	7.67E-12
10, 1	→	11, 0	449.51	1.04E-15	1.47E-14	6.62E-14	3.96E-13	1.53E-12	2.83E-12	5.32E-12	7.61E-12
11, 1	→	12, 0	475.37	6.72E-16	1.17E-14	5.57E-14	3.63E-13	1.46E-12	2.78E-12	5.42E-12	7.77E-12
13, 1	→	14, 0	526.91	3.48E-16	7.18E-15	3.84E-14	2.95E-13	1.33E-12	2.65E-12	5.34E-12	7.63E-12
15, 1	→	16, 0	578.19	2.01E-16	4.40E-15	2.61E-14	2.32E-13	1.17E-12	2.44E-12	5.08E-12	7.24E-12
17, 1	→	18, 0	629.23	8.84E-17	2.71E-15	1.78E-14	1.80E-13	1.01E-12	2.20E-12	4.73E-12	6.79E-12
20, 1	→	21, 0	705.31	3.76E-17	1.34E-15	1.01E-14	1.21E-13	7.97E-13	1.84E-12	4.09E-12	5.95E-12
30, 1	→	31, 0	954.87	5.49E-16	3.20E-16	1.58E-15	2.95E-14	3.00E-13	7.75E-13	1.85E-12	2.96E-12
40, 1	→	41, 0	1198.2	5.21E-15	1.48E-15	1.18E-15	1.43E-14	1.24E-13	3.07E-13	7.88E-13	1.47E-12
1, 0	→	0, 1	−187.46	7.14E-16	1.53E-14	4.09E-14	1.24E-13	3.09E-13	5.12E-13	1.05E-12	1.82E-12
1, 1	→	0, 2	−158.62	3.94E-15	4.86E-14	1.05E-13	2.78E-13	6.74E-13	1.10E-12	2.19E-12	3.64E-12
1, 2	→	0, 3	−129.82	1.56E-14	1.03E-13	1.84E-13	4.52E-13	1.11E-12	1.82E-12	3.51E-12	5.59E-12
1, 4	→	0, 5	−72.37	1.53E-13	3.24E-13	4.99E-13	1.04E-12	2.25E-12	3.53E-12	6.31E-12	9.32E-12
1, 6	→	0, 7	−15.09	8.61E-13	7.69E-13	9.77E-13	1.76E-12	3.52E-12	5.22E-12	8.76E-12	1.24E-11
1, 8	→	0, 9	42.01	9.61E-13	9.45E-13	1.24E-12	2.22E-12	4.29E-12	6.24E-12	1.03E-11	1.42E-11
1, 10	→	0, 11	98.92	6.52E-13	8.00E-13	1.08E-12	2.07E-12	4.26E-12	6.37E-12	1.08E-11	1.50E-11
1, 12	→	0, 13	155.65	2.44E-13	6.29E-13	9.65E-13	1.92E-12	3.98E-12	6.03E-12	1.04E-11	1.47E-11
1, 14	→	0, 15	212.20	8.39E-14	3.62E-13	7.42E-13	1.77E-12	3.75E-12	5.70E-12	9.88E-12	1.39E-11
1, 16	→	0, 17	268.57	3.05E-14	2.01E-13	5.06E-13	1.56E-12	3.60E-12	5.53E-12	9.44E-12	1.28E-11
1, 18	→	0, 19	324.76	1.18E-14	1.11E-13	3.34E-13	1.28E-12	3.39E-12	5.36E-12	9.04E-12	1.20E-11
1, 20	→	0, 21	380.77	5.49E-15	5.90E-14	2.12E-13	1.00E-12	3.11E-12	5.15E-12	8.66E-12	1.13E-11
1, 30	→	0, 31	658.09	1.03E-16	2.93E-15	1.76E-14	1.94E-13	1.28E-12	2.75E-12	4.09E-12	5.23E-12
1, 40	→	0, 41	930.88	2.88E-18	2.27E-16	2.09E-15	3.63E-14	2.97E-13	6.88E-13	1.59E-12	2.81E-12

**Table 6:** V–V rate coefficients (in cm${}^{3}$/s) for the near-resonant asymmetric transition CO$\left(v-2\right)+N_{2}\left(1\right)\to$ CO$\left(v\right)+N_{2}\left(0\right)+\Delta E$.

		$k_{v-2,1\to v,0}$							
*v*	$\Delta E\left({\mathrm{cm}}^{-1}\right)$	100 K	300 K	500 K	1000 K	2000 K	3000 K	5000 K	7000 K
33	−347.21	1.35E-17	9.70E-16	5.71E-15	3.55E-14	1.48E-13	3.43E-13	9.23E-13	1.68E-12
34	−298.17	2.62E-17	2.44E-15	1.16E-14	5.59E-14	1.84E-13	3.81E-13	9.45E-13	1.68E-12
35	−249.26	1.71E-16	6.65E-15	2.49E-14	8.73E-14	2.26E-13	4.24E-13	9.68E-13	1.68E-12
36	−200.47	8.10E-16	1.77E-14	4.89E-14	1.23E-13	2.72E-13	4.76E-13	1.00E-12	1.67E-12
37	−151.81	5.23E-15	4.67E-14	8.72E-14	1.72E-13	3.40E-13	5.49E-13	1.05E-12	1.68E-12
38	−103.28	3.67E-14	1.01E-13	1.43E-13	2.46E-13	4.45E-13	6.60E-13	1.14E-12	1.72E-12
39	−54.87	1.60E-13	2.02E-13	2.42E-13	3.61E-13	5.93E-13	8.07E-13	1.25E-12	1.80E-12
40	−6.58	5.95E-13	3.58E-13	3.61E-13	4.75E-13	7.22E-13	9.05E-13	1.26E-12	1.78E-12
41	41.58	4.53E-13	3.33E-13	3.64E-13	5.12E-13	7.63E-13	8.78E-13	1.09E-12	1.57E-12
42	89.61	2.93E-13	2.61E-13	3.03E-13	4.58E-13	7.17E-13	8.36E-13	1.04E-12	1.49E-12
43	137.53	1.09E-13	1.89E-13	2.45E-13	3.95E-13	6.32E-13	7.44E-13	9.43E-13	1.39E-12
44	185.31	4.59E-14	1.17E-13	1.95E-13	3.49E-13	5.59E-13	6.58E-13	8.56E-13	1.30E-12
45	232.97	1.98E-14	6.88E-14	1.44E-13	3.09E-13	5.04E-13	5.93E-13	7.87E-13	1.22E-12
46	280.51	1.24E-14	4.27E-14	1.01E-13	2.65E-13	4.62E-13	5.43E-13	7.32E-13	1.17E-12

**Table 7:** V–V rate coefficients (in cm${}^{3}$/s) for the near-resonant asymmetric transition CO$\left(v-2\right)+N_{2}\left(2\right)\to$ CO$\left(v\right)+N_{2}\left(1\right)+\Delta E$.

		$k_{v-2,2\to v,1}$							
*v*	$\Delta E\left({\mathrm{cm}}^{-1}\right)$	100 K	300 K	500 K	1000 K	2000 K	3000 K	5000 K	7000 K
37	−180.66	4.04E-15	5.76E-14	1.34E-13	3.07E-13	6.08E-13	9.72E-13	1.95E-12	3.17E-12
38	−132.12	2.35E-14	1.43E-13	2.31E-13	4.33E-13	7.76-13	1.14E-12	2.06E-12	3.23E-12
39	−83.71	1.58E-13	2.78E-13	3.65E-13	5.88E-13	1.02-12	1.41E-12	2.25E-12	3.27E-12
40	−35.42	5.72E-13	5.31E-13	5.98E-13	8.46E-13	1.32-12	1.68E-12	2.40E-12	3.33E-12
41	12.74	1.12E-12	7.38E-13	7.72E-13	1.04E-12	1.44-12	1.66E-12	2.12E-12	2.96E-12
42	60.77	8.47E-13	6.32E-13	7.18E-13	1.07E-12	1.57-12	1.78E-12	2.13E-12	2.86E-12
43	108.68	4.35E-13	4.90E-13	5.94E-13	9.42E-13	1.43-12	1.64E-12	2.00E-12	2.72E-12
44	156.47	1.48E-13	3.34E-13	4.83E-13	8.31E-13	1.27-12	1.45E-12	1.81E-12	2.54E-12

**Table 8:** Single quantum V–T/R rate coefficients (in cm${}^{3}$/s) for the transition CO$\left(v\right)+N_{2}\left(0\right)\to$ CO$\left(v-1\right)+N_{2}\left(0\right)+\Delta E$.

		$k_{v,0\to v-1,0}$							
*v*	$\Delta E\left({\mathrm{cm}}^{-1}\right)$	100 K	300 K	500 K	1000 K	2000 K	3000 K	5000 K	7000 K
1	2143.3	7.19E-16	2.92E-16	2.09E-16	2.27E-16	8.05E-15	8.07E-14	1.15E-12	4.81E-12
3	2090.3	2.93E-15	5.90E-16	2.98E-16	8.91E-16	3.53E-14	3.52E-13	3.83E-12	1.32E-11
5	2037.7	4.88E-15	8.65E-16	4.50E-16	1.88E-15	7.43E-14	6.92E-13	6.64E-12	2.07E-11
7	1985.3	7.04E-15	1.36E-15	6.86E-16	3.02E-15	1.33E-13	1.14E-12	9.65E-12	2.78E-11
10	1907.1	8.39E-15	1.43E-15	7.93E-16	5.76E-15	2.72E-13	2.05E-12	1.47E-11	3.85E-11
12	1855.3	7.43E-15	1.44E-15	8.76E-16	9.50E-15	4.13E-13	2.84E-12	1.86E-11	4.60E-11
15	1778.1	1.24E-14	3.15E-15	1.75E-15	1.96E-14	7.34E-13	4.37E-12	2.49E-11	5.75E-11
17	1727.0	2.21E-14	3.45E-15	2.76E-15	3.24E-14	1.06E-12	5.71E-12	2.95E-11	6.52E-11
20	1650.7	2.99E-14	5.89E-15	3.36E-15	7.33E-14	1.80E-12	8.44E-12	3.77E-11	7.75E-11
25	1524.8	1.89E-13	2.15E-14	1.08E-14	1.88E-13	4.02E-12	1.55E-11	5.48E-11	1.01E-10
30	1400.5	2.04E-13	3.14E-14	1.71E-14	5.25E-13	8.42E-12	2.68E-11	7.55E-11	1.22E-10
35	1277.8	5.16E-13	2.02E-13	1.09E-13	1.31E-12	1.62E-11	4.43E-11	1.07E-10	1.52E-10
40	1156.6	2.72E-12	1.78E-13	1.99E-13	3.23E-12	2.85E-11	6.54E-11	1.30E-10	1.75E-10

**Table 9:** Single quantum V–T/R rate coefficients (in cm${}^{3}$/s) for the transition CO$\left(0\right)+N_{2}\left(v\right)\to$ CO$\left(0\right)+N_{2}\left(v-1\right)+\Delta E$.

		$k_{0,v\to 0,v-1}$							
*v*	$\Delta E\left({\mathrm{cm}}^{-1}\right)$	100 K	300 K	500 K	1000 K	2000 K	3000 K	5000 K	7000 K
1	2330.7	1.92E-19	5.54E-20	1.29E-19	2.37E-17	2.22E-15	2.76E-14	4.78E-13	2.21E-12
3	2273.1	2.36E-18	3.30E-19	1.26E-18	1.10E-16	1.10E-14	1.24E-13	1.59E-12	6.20E-12
5	2215.6	2.21E-17	2.00E-18	2.06E-18	2.34E-16	2.38E-14	2.68E-13	3.09E-12	1.09E-11
7	2158.3	2.73E-17	2.24E-18	4.76E-18	5.27E-16	4.47E-14	4.39E-13	4.51E-12	1.49E-11
10	2072.8	1.54E-17	1.64E-18	9.20E-18	1.21E-15	9.33E-14	8.80E-13	7.90E-12	2.33E-11
12	2015.9	1.84E-17	2.80E-18	1.96E-17	2.42E-15	1.63E-13	1.27E-12	1.04E-11	2.89E-11
15	1931.1	3.41E-17	5.71E-18	4.55E-17	4.76E-15	2.72E-13	2.09E-12	1.50E-11	3.85E-11
17	1874.7	1.22E-17	3.79E-18	8.12E-17	7.94E-15	4.01E-13	2.83E-12	1.86E-11	4.57E-11
20	1790.5	8.19E-18	7.65E-18	1.96E-16	1.67E-14	6.97E-13	4.29E-12	2.47E-11	5.70E-11
25	1651.0	5.33E-18	2.80E-17	8.13E-16	5.49E-14	1.66E-12	8.07E-12	3.68E-11	7.57E-11
30	1512.7	4.24E-18	1.27E-16	3.28E-15	1.73E-13	3.88E-12	1.53E-11	5.49E-11	9.91E-11
35	1375.5	1.13E-17	6.44E-16	1.30E-14	5.22E-13	8.42E-12	2.65E-11	7.37E-11	1.18E-10
40	1239.5	1.95E-17	3.09E-15	5.14E-14	1.51E-12	1.70E-11	4.41E-11	1.00E-10	1.47E-10

**Table 10:** Multiquantum V–T/R rate coefficients (in cm${}^{3}$/s) for the transition CO$\left(v\right)+N_{2}\left(0\right)\to$ CO$\left(v^{\prime }\right)+N_{2}\left(0\right)+\Delta E$.

			$k_{v,0\to v^{\prime },0}$							
*v*	$v^{\prime }$	$\Delta E\left({\mathrm{cm}}^{-1}\right)$	100K	300 K	500 K	1000 K	2000 K	3000 K	5000 K	7000 K
3	1	4207.1	1.79E-17	3.40E-18	1.49E-18	1.36E-18	2.85E-16	7.66E-15	2.11E-13	1.22E-12
3	0	6350.4	1.56E-19	2.15E-20	9.20E-21	5.99E-21	3.02E-18	1.51E-16	1.10E-14	1.08E-13
5	3	4101.7	4.81E-17	1.02E-17	4.50E-18	4.49E-18	1.28E-15	3.05E-14	6.71E-13	3.18E-12
5	2	6192.0	1.20E-18	1.66E-19	7.10E-20	5.26E-20	4.04E-17	1.65E-15	7.64E-14	5.58E-13
7	5	3996.7	2.23E-16	2.65E-17	1.03E-17	1.18E-17	3.61E-15	7.48E-14	1.32E-12	5.40E-12
7	4	6034.4	3.72E-18	4.95E-19	2.10E-19	1.96E-19	1.83E-16	6.05E-15	1.85E-13	1.08E-12
10	8	3840.2	3.25E-16	4.95E-17	1.90E-17	3.23E-17	1.13E-14	1.90E-13	2.63E-12	9.30E-12
10	7	5799.4	9.77E-18	1.27E-18	4.85E-19	6.93E-19	8.59E-16	2.23E-14	4.77E-13	2.18E-12
12	10	3736.5	3.92E-16	6.59E-17	2.66E-17	6.55E-17	2.07E-14	3.08E-13	3.69E-12	1.22E-11
12	9	5643.7	1.93E-17	2.49E-18	9.68E-19	1.68E-18	1.90E-15	4.27E-14	7.75E-13	3.19E-12
15	13	3581.9	1.06E-15	1.45E-16	4.89E-17	1.97E-16	4.54E-14	5.68E-13	5.61E-12	1.67E-11
15	12	5411.5	5.07E-17	5.36E-18	1.92E-18	6.16E-18	5.30E-15	9.79E-14	1.42E-12	5.23E-12
17	15	3479.5	1.16E-15	1.91E-16	8.70E-17	4.22E-16	7.26E-14	8.07E-13	7.12E-12	2.00E-11
17	14	5257.6	6.24E-17	1.23E-17	4.98E-18	1.50E-17	9.76E-15	1.59E-13	2.00E-12	6.88E-12
20	18	3326.8	3.68E-15	3.91E-16	2.29E-16	1.21E-15	1.41E-13	1.29E-12	9.70E-12	2.52E-11
20	17	5028.2	8.37E-17	3.48E-17	7.95E-18	5.28E-17	2.20E-14	3.02E-13	3.13E-12	9.82E-12
25	23	3074.7	2.68E-15	4.89E-16	2.31E-16	6.57E-15	4.22E-13	2.79E-12	1.60E-11	3.67E-11
25	22	4649.7	4.05E-16	5.11E-17	1.89E-17	4.04E-16	7.41E-14	7.61E-13	5.92E-12	1.59E-11
30	28	2825.8	6.08E-15	6.98E-16	5.39E-16	3.38E-14	1.33E-12	6.42E-12	2.72E-11	5.34E-11
30	27	4275.8	5.09E-16	8.39E-17	4.75E-17	4.02E-15	3.17E-13	2.18E-12	1.25E-11	2.79E-11
35	33	2580.0	5.76E-14	2.85E-15	7.10E-15	1.25E-13	3.07E-12	1.17E-11	4.03E-11	7.33E-11
35	32	3906.7	1.66E-15	2.28E-16	2.20E-16	1.69E-14	7.57E-13	3.71E-12	1.74E-11	3.78E-11
40	38	2337.3	4.65E-14	5.28E-15	8.40E-15	4.94E-13	7.82E-12	2.66E-11	5.87E-11	9.20E-11
40	37	3542.2	5.22E-15	6.35E-16	1.33E-15	1.21E-13	2.77E-12	9.91E-12	3.13E-11	5.47E-11

**Table 11:** Multiquantum V–T/R rate coefficients (in cm${}^{3}$/s) for the transition CO$\left(0\right)+N_{2}\left(v\right)\to$ CO$\left(0\right)+N_{2}\left(v^{\prime }\right)+\Delta E$.

			$k_{0,v\to 0,v^{\prime }}$							
*v*	$v^{\prime }$	$\Delta E\left({\mathrm{cm}}^{-1}\right)$	100 K	300 K	500 K	1000 K	2000 K	3000 K	5000 K	7000 K
3	1	4574.9	9.15E-20	1.44E-20	1.04E-20	3.65E-20	3.27E-17	1.20E-15	4.90E-14	3.45E-13
3	0	6905.7	6.65E-21	1.07E-21	5.09E-22	1.74E-22	1.54E-19	1.42E-17	1.42E-15	1.67E-14
5	3	4459.9	2.93E-19	4.66E-20	2.04E-20	1.30E-19	1.98E-16	5.73E-15	1.77E-13	1.08E-12
5	2	6733.0	6.41E-20	8.78E-21	3.27E-21	1.22E-21	2.63E-18	1.52E-16	1.25E-14	1.22E-13
7	5	4345.3	3.77E-19	5.72E-20	3.02E-20	1.30E-19	4.16E-16	1.14E-14	2.96E-13	1.57E-12
7	4	6560.9	1.32E-19	1.98E-20	8.27E-21	1.22E-21	5.14E-18	2.65E-16	1.21E-14	1.81E-13
10	8	4174.0	5.97E-19	1.18E-19	5.00E-20	5.89E-18	2.02E-15	3.94E-14	8.68E-13	3.84E-12
10	7	6303.8	2.93E-19	2.94E-20	1.02E-20	5.12E-20	4.84E-17	2.32E-15	1.04E-13	7.00E-13
12	10	4060.3	2.97E-19	6.50E-20	4.96E-20	5.40E-18	4.38E-15	8.52E-14	1.35E-12	5.37E-12
12	9	6133.0	1.84E-19	3.28E-20	1.33E-20	6.60E-20	1.91E-16	5.81E-15	1.79E-13	1.08E-12
15	13	3890.4	3.07E-19	8.04E-20	7.41E-20	1.93E-17	1.11E-14	1.84E-13	2.39E-12	8.32E-12
15	12	5878.0	1.91E-19	4.00E-20	1.71E-20	3.18E-19	6.36E-16	1.58E-14	3.63E-13	1.86E-12
17	15	3777.5	2.10E-19	7.30E-20	1.11E-19	4.36E-17	1.92E-14	2.88E-13	3.34E-12	1.08E-11
17	14	5708.6	1.87E-19	4.82E-20	2.09E-20	8.46E-19	1.29E-15	2.88E-14	5.61E-13	2.60E-12
20	18	3609.0	4.12E-19	5.86E-20	2.99E-19	2.09E-16	4.10E-14	4.97E-13	5.43E-12	1.65E-11
20	17	5455.5	2.58E-19	3.76E-20	1.96E-20	5.44E-18	3.38E-15	6.72E-14	1.15E-12	4.39E-12
25	23	3329.8	4.50E-19	8.18E-20	2.40E-18	1.03E-15	1.70E-13	1.59E-12	1.15E-11	2.86E-11
25	22	5036.5	1.55E-19	2.59E-20	4.19E-20	3.16E-17	1.92E-14	2.60E-13	2.90E-12	9.26E-12
30	28	3053.0	1.59E-18	3.73E-19	1.94E-17	6.76E-15	5.98E-13	3.75E-12	2.03E-11	4.33E-11
30	27	4620.8	2.61E-19	3.68E-20	3.62E-19	5.93E-16	1.13E-13	1.07E-12	8.40E-12	2.13E-11
35	33	2778.4	1.55E-17	3.00E-18	1.40E-16	2.67E-14	1.23E-12	6.10E-12	2.60E-11	5.09E-11
35	32	4208.6	1.55E-19	6.59E-20	4.37E-18	2.65E-15	2.91E-13	2.14E-12	1.23E-11	2.74E-11
40	38	2506.0	3.33E-18	1.05E-16	1.09E-15	1.29E-13	3.40E-12	1.24E-11	3.93E-11	6.77E-11
40	37	3799.8	7.98E-20	4.46E-19	5.84E-17	1.81E-14	8.82E-13	4.49E-12	1.96E-11	3.84E-11

**Table 12:** V–V and V–T/R rate coefficients (in cm${}^{3}$/s) for the transitions CO$\left(v_{1}\right)+N_{2}\left(v_{2}\right)\to$ CO$\left(v_{1}^{\prime }\right)+N_{2}\left(v_{2}^{\prime }\right)+\Delta E$.

				$k_{v_{1}v_{2}\to v_{1}^{\prime }v_{2}^{\prime }}$							
$v_{1}v_{2}$	→	$v_{1}^{\prime }v_{2}^{\prime }$	$\Delta E\left({\mathrm{cm}}^{-1}\right)$	100 K	300 K	500 K	1000 K	2000 K	3000 K	5000 K	7000 K
10 10	→	9 11	−137.21	3.87E-13	2.98E-12	5.27E-12	1.12E-11	2.40E-11	3.65E-11	5.67E-11	6.96E-11
10 10	→	11 9	191.57	8.98E-13	3.12E-12	5.58E-12	1.13E-11	2.25E-11	3.33E-11	5.10E-11	6.26E-11
10 10	→	10 9	2072.8	2.85E-17	1.04E-17	2.04E-17	1.72E-15	9.83E-14	6.98E-13	4.61E-12	1.15E-11
10 10	→	9 10	1907.1	4.52E-15	9.85E-16	6.48E-16	4.82E-15	1.99E-13	1.34E-12	8.45E-12	2.05E-11
20 20	→	19 21	−111.79	2.98E-12	1.26E-11	1.89E-11	3.39E-11	6.04E-11	8.15E-11	1.05E-10	1.33E-10
20 20	→	21 19	165.07	4.42E-12	1.31E-11	1.95E-11	3.25E-11	5.50E-11	7.36E-11	9.51E-11	1.02E-10
20 20	→	20 19	1790.5	4.16E-16	1.21E-16	4.21E-16	2.49E-14	6.73E-13	2.88E-12	1.14E-11	2.23E-11
20 20	→	19 20	1650.7	2.17E-14	2.61E-15	2.80E-15	4.30E-14	1.01E-12	4.36E-12	1.69E-11	3.23E-11

**Table 13:** Multiquantum V–T/R rate coefficients (in cm${}^{3}$/s) for the transition CO$\left(1\right)+N_{2}\left(v\right)\to$ CO$\left(1\right)+N_{2}\left(v^{\prime }\right)+\Delta E$.

			$k_{0,v\to 0,v^{\prime }}$							
*v*	$v^{\prime }$	$\Delta E\left({\mathrm{cm}}^{-1}\right)$	100 K	300 K	500 K	1000 K	2000 K	3000 K	5000 K	7000 K
1	0	2330.7	1.95E-17	2.29E-18	7.91E-19	2.26E-17	2.78E-15	3.42E-14	4.42E-13	1.74E-12
5	4	2215.6	1.61E-17	1.88E-18	3.09E-18	2.61E-16	2.38E-14	2.43E-13	2.60E-12	8.73E-12
5	3	4459.9	1.71E-19	3.14E-20	2.99E-20	1.90E-19	1.42E-16	4.08E-15	1.12E-13	6.10E-13
5	2	6733.0	4.06E-20	7.49E-21	3.96E-21	1.46E-21	1.21E-18	6.85E-17	3.55E-15	3.07E-14
10	9	2072.8	9.89E-18	2.89E-18	1.35E-17	1.51E-15	9.59E-14	7.51E-13	6.46E-12	1.94E-11
10	8	4174.0	1.59E-19	7.95E-20	5.44E-20	3.66E-18	1.29E-15	2.40E-14	4.68E-13	2.12E-12
10	7	6303.8	1.73E-19	2.97E-20	1.07E-20	4.11E-20	1.78E-17	6.01E-16	2.17E-14	1.46E-13
20	19	1790.5	4.82E-17	1.17E-17	2.33E-16	1.89E-14	7.63E-13	4.27E-12	2.31E-11	5.20E-11
20	18	3609.0	5.07E-19	9.27E-20	3.82E-19	2.37E-16	3.91E-14	4.33E-13	4.48E-12	1.36E-11
20	17	5455.5	9.81E-20	1.47E-20	9.41E-21	5.80E-18	2.94E-15	5.39E-14	8.83E-13	3.42E-12
30	29	1512.7	1.51E-16	1.71E-16	3.14E-15	1.74E-13	3.92E-12	1.51E-11	5.60E-11	1.03E-10
30	28	3053.0	5.82E-19	3.55E-19	2.33E-17	5.21E-15	3.48E-13	2.59E-12	1.66E-11	3.77E-11
30	27	4620.8	1.21E-19	5.33E-20	5.83E-19	2.39E-16	4.37E-14	5.24E-13	5.08E-12	1.40E-11
40	39	1239.5	1.82E-17	3.46E-15	4.80E-14	1.45E-12	1.80E-11	4.72E-11	1.04E-10	1.47E-10
40	38	2506.0	3.83E-19	1.92E-17	1.18E-15	1.52E-13	3.68E-12	1.28E-11	4.00E-11	6.78E-11
40	37	3799.8	8.41E-20	6.58E-19	1.02E-16	2.16E-14	8.28E-13	4.34E-12	2.10E-11	4.18E-11

**Table 14:** Multiquantum V–T/R rate coefficients (in cm${}^{3}$/s) for the transition CO$\left(v\right)+N_{2}\left(1\right)\to$ CO$\left(v^{\prime }\right)+N_{2}\left(1\right)+\Delta E$.

			$k_{0,v\to 0,v^{\prime }}$							
*v*	$v^{\prime }$	$\Delta E\left({\mathrm{cm}}^{-1}\right)$	100 K	300 K	500 K	1000 K	2000 K	3000 K	5000 K	7000 K
1	0	2143.3	1.06E-15	3.01E-16	1.51E-16	1.94E-16	8.82E-15	9.61E-14	1.17E-12	4.41E-12
5	4	2037.7	4.76E-15	8.80E-16	4.62E-16	1.87E-15	7.23E-14	6.63E-13	6.20E-12	1.89E-11
5	3	4101.7	5.01E-17	1.00E-17	4.38E-18	4.42E-18	1.20E-15	2.78E-14	5.69E-13	2.55E-12
5	2	6192.0	1.11E-18	1.55E-19	6.73E-20	5.23E-20	3.72E-17	1.42E-15	5.61E-14	3.68E-13
10	9	1907.1	8.01E-15	1.40E-15	8.84E-16	6.92E-15	2.88E-13	2.05E-12	1.40E-11	3.55E-11
10	8	3840.2	2.23E-16	4.68E-17	2.39E-17	5.39E-17	9.46E-15	1.51E-13	2.05E-12	7.37E-12
10	7	5799.4	1.34E-17	1.74E-18	9.55E-19	1.11E-18	4.82E-16	1.37E-14	3.37E-13	1.67E-12
20	19	1650.7	3.96E-14	5.36E-15	3.54E-15	6.60E-14	1.91E-12	9.09E-12	3.93E-11	7.80E-11
20	18	3326.8	1.47E-15	2.85E-16	1.29E-16	1.61E-15	1.65E-13	1.34E-12	9.29E-12	2.35E-11
20	17	5028.2	1.79E-16	2.36E-17	9.94E-18	7.54E-17	1.57E-14	2.05E-13	2.35E-12	7.80E-12
30	29	1400.5	3.62E-13	6.04E-14	3.52E-14	4.92E-13	8.68E-12	2.75E-11	7.90E-11	1.31E-10
30	28	2825.8	3.93E-14	4.37E-15	1.66E-15	3.41E-14	1.28E-12	5.86E-12	2.62E-11	5.33E-11
30	27	4275.8	4.97E-15	3.73E-16	1.22E-16	3.32E-15	2.15E-13	1.56E-12	1.05E-11	2.56E-11
40	39	1156.6	4.99E-12	8.57E-13	4.08E-13	3.38E-12	2.94E-11	6.62E-11	1.31E-10	1.79E-10
40	38	2337.3	6.98E-13	3.97E-14	1.88E-14	5.72E-13	8.71E-12	2.42E-11	5.93E-11	9.04E-11
40	37	3542.2	3.98E-14	4.42E-15	2.57E-15	1.38E-13	3.09E-12	1.03E-11	3.16E-11	5.46E-11

## Data Availability

Not applicable.

## Supplementary Materials

The following are available online, Figure S1: Landau–Teller plot of the rate coefficients for the transition (40,0) → (38,1).

## Sample Availability

Samples of the compounds are not available from the authors.

## Appendix A. The Quantum-Classical Method

Solving the dynamical equations of motion can be done either by adopting a quantum mechanical or a classical mechanical viewpoint. For classical simulations the most common approach uses QCT method, the limits of which have been found at relatively low energy and with low atomic masses when the classical mechanical description appears to be insufficient [56]. On the other side, accurate quantum mechanical calculations have been used both for time-dependent and time-independent calculations for many light particle systems [57], but these kinds of simulation are still time-consuming and prohibitive for heavy particle having thousands of rovibrational states. Since the various degrees of freedom are more or less classical, a feasible way to solve the problem is to mix quantum and classical mechanics. The quantum-classical method introduced and developed by Billing [7,9], in which only the vibrational motion is quantized, is able to produce cross sections for heavy particle collisions in a reasonable amount of time and with good accuracy.

The simplifying assumption of the method is that the rotations of both molecules are treated classically as is the relative translational motion, thus only the vibrational degrees of freedom are quantized. A brief description of the theory of the method follows; for more detailed information readers are referred to Refs. [7,9].

In the present quantum-classical method for two diatomic molecules, the vibrations and the rotational-vibrational coupling are treated quantum-mechanically by close coupled equations. First of all, the total vibrational wavefunction is expanded in terms of the product of rotationally perturbed Morse wave functions $\phi_{v_{1}}\left(r_{1},t\right)\phi_{v_{2}}\left(r_{2},t\right)$ as follows:

(A1) $$\Psi \left(r_{1},r_{2},t\right)=\sum_{v_{1}v_{2}}a_{v_{1}v_{2}}\left(t\right)\phi_{v_{1}}\left(r_{1},t\right)\phi_{v_{2}}\left(r_{2},t\right)\exp\left[-\frac{it\left(E_{v_{1}}+E_{v_{2}}\right)}{\hbar }\right],$$

where $a_{v_{1}v_{2}}\left(t\right)$ is the expansion coefficient, $r_{i}$ is the intramolecular distance of diatom *i*, and $E_{v_{i}}$ is the eigenvalue of the rotationally perturbed Morse wave functions $\phi_{v_{i}}\left(r_{i},t\right)$, which depends upon time through the rotational-vibrational coupling terms:

(A2) $$\phi_{v_{i}}\left(r_{i},t\right)=\phi_{v_{i}}^{0}\left(r_{i}\right)+\sum_{v_{i}^{\prime }\neq v_{i}}\phi_{v_{i}^{\prime }}^{0}\left(r_{i}\right)\frac{H_{v_{i}^{\prime }v_{i}}}{E_{v_{i}}^{0}-E_{v_{i}^{\prime }}^{0}},$$

where $H_{v_{i}^{\prime }v_{i}}$ is the first-order centrifugal stretching term:

(A3) $$H_{v_{i}^{\prime }v_{i}}=-j_{i}^{2}m_{i}^{-1}{\bar{r}}_{i}^{-3}<\phi_{v_{i}^{\prime }}^{0}\left|r_{i}-{\bar{r}}_{i}\right|\phi_{v_{i}}^{0}>$$

with $j_{i}$ being the rotational momentum of molecule *i* and the operator <> is obtained by integrating over $r_{i}$. $\phi_{v_{i}}^{0}$ is the unperturbed eigenfunction of the Morse oscillator and $E_{v_{i}}^{0}$ is the eigenvalue approximated as

(A4) $$E_{v_{i}}^{0}=\hbar \omega_{e}\left(v_{i}+\frac{1}{2}\right)-\hbar \omega_{e}x_{e}{\left(v_{i}+\frac{1}{2}\right)}^{2}+\hbar \omega_{e}y_{e}{\left(v_{i}+\frac{1}{2}\right)}^{3},$$

where $\omega_{e}$ is the oscillator wavenumber and $x_{e}$ and $y_{e}$ are the anharmonicity constants.

In order to solve for the expansion coefficients in Equation (A1), one can plug the expansion into the time-dependent Schrödinger equation and obtain the following set of equations:

(A5) $$\begin{array}{c} i\hbar {\dot{a}}_{v_{1}^{\prime }v_{2}^{\prime }}\left(t\right)=\sum_{v_{1}v_{2}}\left(\begin{array}{c} \delta_{v_{2}v_{2}^{\prime }}M_{v_{1}^{\prime }v_{1}}^{\left(1\right)}A^{\left(1\right)}+\delta_{v_{1}v_{1}^{\prime }}M_{v_{2}^{\prime }v_{2}}^{\left(1\right)}A^{\left(2\right)}\\ +\frac{1}{2}\delta_{v_{2}v_{2}^{\prime }}M_{v_{1}^{\prime }v_{1}}^{\left(2\right)}B^{\left(1\right)}+\frac{1}{2}\delta_{v_{1}v_{1}^{\prime }}M_{v_{2}^{\prime }v_{2}}^{\left(2\right)}B^{\left(2\right)}+M_{v_{1}^{\prime }v_{1}}^{\left(1\right)}M_{v_{2}^{\prime }v_{2}}^{\left(1\right)}C \end{array}\right)\\ \cdot a_{v_{1}v_{2}}\left(t\right)\exp\left[\frac{i}{\hbar }\left(E_{v_{1}^{\prime }}+E_{v_{2}^{\prime }}-E_{v_{1}}-E_{v_{2}}\right)t\right], \end{array}$$

where

(A6) $$\begin{array}{c} A^{\left(i\right)}={\left.\frac{\partial V}{\partial r_{i}}\right|}_{eq}+2i\hbar j_{i}\frac{dj_{i}}{dt}\frac{1}{m_{i}r_{eq,i}^{3}\left(E_{v_{i}}^{0}-E_{v_{i}^{\prime }}^{0}\right)}, \end{array}$$

(A7) $$\begin{array}{c} B^{\left(i\right)}={\left.\frac{\partial^{2}V}{\partial r_{i}^{2}}\right|}_{eq}, \end{array}$$

(A8) $$\begin{array}{c} C={\left.\frac{\partial^{2}V}{\partial r_{1}\partial r_{2}}\right|}_{eq}, \end{array}$$

and

(A9) $$\begin{array}{c} M_{v_{i}^{\prime }v_{i}}^{\left(k\right)}=\langle \phi_{v_{i}^{\prime }}^{0}\left|{\left(r_{i}-r_{i,eq}\right)}^{k}\right|\phi_{v_{i}}^{0}\rangle . \end{array}$$

The classical dynamics, driven by an averaged Ehrenfest potential [8] $V_{eff}$, is used for the relative translational motion and the rotational motions of both molecules. To be more specific, the classical Hamiltonian is

(A10) $$\begin{array}{cc} H_{cl}= & \frac{1}{2\mu }\left(P_{X}^{2}+P_{Y}^{2}+P_{Z}^{2}\right)+\sum_{i=1,2}\left[\frac{1}{2m_{i}}\left(p_{x_{i}}^{2}+p_{y_{i}}^{2}+p_{z_{i}}^{2}\right)-\lambda_{i}\left({\left(r_{eq}^{i}\right)}^{2}-x_{i}^{2}-y_{i}^{2}-z_{i}^{2}\right)\right]\\ \; & +V_{eff}, \end{array}$$

in which the first term is the kinetic energy of the relative motion, and the second one the rotational motion of the two diatomic molecules; a Lagrange multiplier (third term) is introduced to keep the bond length fixed. Finally, the effective potential is obtained as the expectation value of the total interaction potential over the total wave function $\Psi \left(r_{1},r_{2},t\right)$, thus the dependence of the quantum coordinates is averaged out.

The classical Hamilton equations and the coupled equations (Equation (A5)) are solved by a variable-order variable-step Adams predictor-corrector integrator [58]. An absolute integration accuracy of $10^{-8}$ is achieved for all calculations in this work. The vibrational wavefunction is initialized as the product of the Morse-oscillator wave functions for the two infinitely separated diatoms. The cross-sections are obtained by averaging over a number of trajectories having randomly selected initial conditions, and a Monte Carlo average over the initial Boltzmann distribution of rotational energy is generally introduced to have rate coefficients for vibrational relaxation. Specifically an averaged cross-section is defined as:

(A11) $$\begin{array}{cc} \sigma_{v_{1}v_{2}\to v_{1}^{\prime }v_{2}^{\prime }}\left(U,T_{0}\right)= & \frac{\pi \hbar^{6}}{8\mu k_{B}^{3}T_{0}^{3}I_{1}I_{2}}\int_{0}^{l_{\max}}\int_{0}^{j_{1max}}\int_{0}^{j_{2max}}dj_{1}dj_{2}dl\\ & \cdot \left(2j_{1}+1\right)\left(2j_{2}+1\right)\left(2l+1\right)P_{v_{1}v_{2}\to v_{1}^{\prime }v_{2}^{\prime }} \end{array},$$

where $P_{v_{1}v_{2}\to v_{1}^{\prime }v_{2}^{\prime }}$ is the probability of the process $\mathrm{CO}\left(v_{1}\right)+N_{2}\left(v_{2}\right)\to \mathrm{CO}\left(v_{1}^{\prime }\right)+N_{2}\left(v_{2}^{\prime }\right)$, μ is the reduced mass for the relative motion, and *l* is the orbital angular momentum. The moment of inertia is $I_{i}=m_{i}r_{i}^{2}$ and the temperature $T_{0}$ is arbitrary because it cancels when calculating the rate coefficients. $j_{1max}$ and $j_{2max}$ are the upper limit for the randomly chosen rotational quantum numbers for the diatoms (CO and N${}_{2}$ respectively) and $l_{max}$ is the upper limit for the orbital angular momentum. Rate coefficients are then calculated through the following equation

(A12) $$\begin{array}{cc} k_{v_{1}v_{2}\to v_{1}^{\prime }v_{2}^{\prime }}\left(T\right) & ={\left(\frac{8k_{B}T}{\pi \mu }\right)}^{1/2}{\left(\frac{T_{0}}{T}\right)}^{3}\int_{0}^{\infty }d\left(\frac{\bar{U}}{k_{B}T}\right)\\ & \cdot \exp\left(-\frac{\bar{U}}{k_{B}T}\right)\sigma_{v_{1}v_{2}\to v_{1}^{\prime }v_{2}^{\prime }}\left(T_{0},\bar{U}\right), \end{array}$$

which holds for exothermic processes. The symmetrized classical energy $\bar{U}$ is introduced to restore detailed balance principle [7,8].

## Appendix B. Analytical Rate Coefficients Based on SSH Theory

The analytical approximation for the rate coefficients of CO$\left(v\right)+N_{2}\left(u\right)\to$ CO$\left(v-1\right)+N_{2}\left(u+1\right)$ and CO$\left(v\right)+N_{2}\left(u\right)\to$ CO$\left(v+1\right)+N_{2}\left(u-1\right)$ processes was made by using the well-known expression of the modified SSH theory and includes two components corresponding to the short-range and long-range parts of the interaction potential [11,59], i.e.,

(A13) $$\begin{array}{c} k\left(v,u|v-1,u+1;T\right)\;=a\cdot Z\cdot T\cdot \frac{v}{\left(1-v\cdot \delta \right)}\cdot \frac{\left(u+1\right)}{\left(1-(u+1)\cdot \delta \right)}\cdot \exp\left(\frac{\Delta E}{2kT}\right)\cdot f\left(y\right)\cdot F\\ +b\cdot \frac{Z}{T}\cdot \frac{v}{\left(1-v\cdot \delta \right)}\cdot \frac{\left(u+1\right)}{\left(1-(u+1)\cdot \delta \right)}\cdot \exp\left(\frac{\Delta E}{2kT}\right)\cdot \exp\left(-\frac{\Delta E^{2}}{CkT}\right) \end{array},$$

where $a=2.60\times 10^{-7}\;K$, b=0.07K, and C=165K, which are fitted to the present QC results by minimizing the quadratic error. Moreover, *Z* is the gas kinetic collision rate coefficient, δ is the anharmonicity factor and f(y) is defined as

(A14) $$\begin{array}{c} f(y)=0.5\cdot \exp(-2y/3)\cdot (3-\exp(-2y/3)), \end{array}$$

with

(A15) $$\begin{array}{c} y=l_{\mathrm{ST}}\cdot \left|\Delta E\right|\cdot \pi^{2}\cdot \sqrt{\frac{2\cdot \mu }{h^{2}\cdot k\cdot T}}, \end{array}$$

where μ is the reduced mass of the collision partners and the short range parameter $l_{\mathrm{ST}}$ is tuned to be 0.233 Å. The above best-fitted $l_{\mathrm{ST}}$ is larger than the one proposed in ref. [11], this is because the present rates are higher (see Table 4) due to the more repulsive short range character of the present PES. Furthermore,

(A16) $$\begin{array}{c} F=\exp\left[\frac{4}{\pi \cdot \sqrt{T^{*}}}\cdot {\left(y\right)}^{1/3}+\frac{16}{3\cdot \pi^{2}\cdot T^{*}}\right], \end{array}$$

with $T^{*}=\frac{T}{\varepsilon_{ST}}$ and $\varepsilon_{\mathrm{ST}\;}=125$ K (being equal to the Lennard–Jones potential well depth of the title system).
