# Supplementary material for: Vibrational Energy Transfer in CO+N2 Collisions: A Database for V–V and V–T/R Quantum-Classical Rate Coefficients
Source: Molecules. 2021 Nov 25;26(23):7152. doi: 10.3390/molecules26237152 (PMC8659027; doi:10.3390/molecules26237152)
Supplement: Supplementary file 1 [file molecules-26-07152-s001.zip › molecules-1471078-supplementary.pdf]

Supplementary Materials: Vibrational Energy Transfer in $CO+N_2$ Collisions: a Database for V-V and V-T/R Quantum-Classical Rate Coefficients

Qizhen Hong [1,2], Massimiliano Bartolomei [3], Cecilia Coletti [4*], Andrea Lombardi [5], Quanhua Sun [1,2] and Fernando Pirani [5]

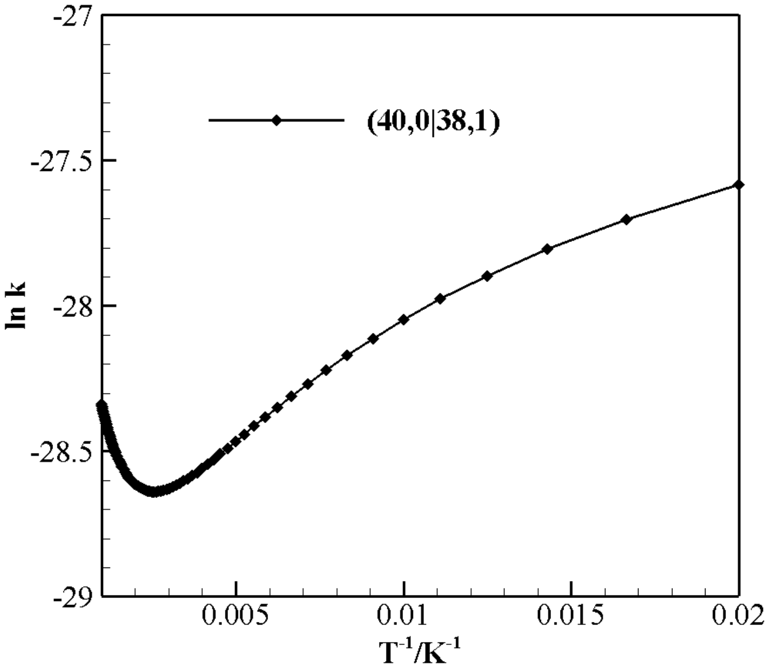

Figure S1. Landau-Teller plot of the rate coefficients for the transition (40,0)⟶(38,1). An anti-Arrhenius behavior is found at low temperature.
